# How the Variability of Iron-Polyphenolic Complexes Affects the Degradation of Iron-Gall Inks: A Multi-Analytical Study

**DOI:** 10.1021/acsomega.5c09386

**Published:** 2026-01-21

**Authors:** Salvatore Caterino, Iulia-Maria Caniola, Marc Pignitter, Alfonso Zoleo, Santiago Sanchez-Cortés, Katja Sterflinger, Federica Cappa

**Affiliations:** † Institute of Natural Sciences and Technology in the Arts, Academy of Fine Arts of Vienna, Vienna 1010, Austria; ‡ Institute of Physiological Chemistry, Faculty of Chemistry, University of Vienna, Vienna 1090, Austria; § Department of Chemical Sciences, University of Padua, Padua 35131, Italy; ∥ Instituto de Estructura de la Materia (CSIC), Madrid 28006, Spain

## Abstract

Iron-gall inks (IGI) were among the most widely used writing materials in historical manuscripts. However, their presence is now recognized as a major cause of degradation in many of these documents. Common forms of deterioration include ink fading and discoloration, embrittlement of the writing support, crack formation, and material loss. Understanding the mechanisms underlying IGI-induced degradation is therefore crucial for developing effective strategies for the preservation of historically valuable manuscripts. In this study, a systematic and multi-analytical approach, involving the use of Raman, electron paramagnetic resonance, and infrared spectroscopy, was employed to investigate the degradation processes associated with IGI, with a specific focus on their intrinsic chemical variability. Three key parameters were considered: the structure of the polyphenolic ligand, the pH, and the iron-to-ligand ratio. These variables were selected to evaluate their respective contributions to the observed degradation phenomena. The findings provide a comprehensive overview of the main degradation pathways and the factors influencing them. Among the results, some indicate the occurrence of hydrolytic processes involving the complex ligands, which seem confined to the acidic conditions applied during sample preparation. In addition, the results enabled comparison of the differences in oxidation rates observed during accelerated aging, revealing how these rates vary according to the structure of the complexes. Overall, the study establishes a robust and reproducible framework that lays the groundwork for future research on IGI-related degradation.

## Graphical abstract

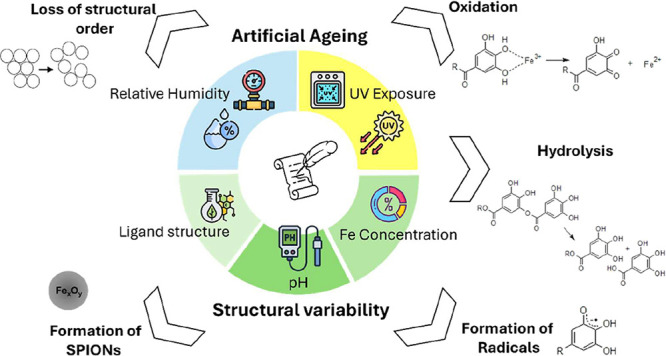

## 1 Introduction

Iron gall inks (IGI) were among the most commonly used writing materials in ancient times. They were particularly popular during the Middle Ages, especially in the centuries XII-XIX, even if there are records of their use also in previous times.^1,2^ In the last decades, a special interest in the scientific investigation of IGI has been manifested. One of the main reasons for that is the strong degradation of IGI-containing manuscripts. The presence of IGI can in fact be associated with severe damage which compromise the state of conservation of a large number of documents. The types of damage range from the formation of haloes and discoloration to the embrittlement of the support, up to the formation of actual holes.^3^

Due to their large diffusion, a great number of historical recipes can be encountered in ancient manuscripts.^1,4^ However, the preparation of such inks generally included the use of at least three main ingredients: a botanical extract rich in polyphenols, a source of iron cations and an organic binder. The most mentioned vegetal matrices used in the extract preparation were oak galls (OG), especially the ones from Aleppo oak (*Quercus infectoria*), from which the polyphenols were extracted using mainly water, even if sometimes wine or other aqueous mixtures were used instead of water.^1,4–6^ As source of iron cations, and in particular ferrous cations, green vitriol (FeSO_4_·7H_2_O) was often used.^1^ Finally, Arabic gum (AG) constitutes the most common organic binder mentioned in historical recipes.^1,5,6^

From a chemical perspective, the dark-bluish ink is formed due to the interaction between the Fe­(II) cations and the polyphenols, mainly medium- to low-molecular-weight tannins, present in the extract, which leads to the formation of insoluble Fe­(III)-polyphenolic complexes via a double step mechanism.^7^

Initially, Fe­(II) cations are coordinated by the polyphenols present in the OG extract, resulting in an immediate formation of dark soluble complexes.^1,7–9^ This step has been proven to be strongly pH-sensitive. On one hand, the complexation itself results in a drop in pH down to levels in the range of 2–3.5.^5,10,11^ However, the process not only influences but is also influenced by the pH. On the other hand in fact, the pH dictates the metal-to-ligand stoichiometry, influencing the protonation/deprotonation state of the polyphenolic ligands.^7,8,12–18^ The second step is the so-called “auto-oxidation”: the soluble Fe­(II)-polyphenolic complexes are converted into insoluble Fe­(III)-polyphenolic complexes via oxidation with atmospheric oxygen.^5,8,19,20^

In a previous study, the role of the pH (prior to the iron salt addition), polyphenols’ structure and iron concentration in defining the final structure of these complexes was investigated. Throughout the use of mainly Raman and EPR spectroscopy, it has been demonstrated that the pH is the most affecting parameter in the formation of Fe­(III)-polyphenolic complexes, influencing not only the stoichiometric ratios between metal and ligands but also dictating the geometry of the coordination.^7,8,19^ Moreover, thanks to the accurate experimental design implemented for that study, it has been possible to efficiently distinguish between Fe­(III) complexes prepared using different polyphenolic ligands just via non-invasive techniques such as Raman and infrared (IR) spectroscopies. The current study makes a step further in investigating the role of these same three important variables, namely pH, polyphenols’ structure, and iron concentration, in the degradation processes affecting IGI. The aim of this research is to gain deeper insight into the degradation mechanisms intrinsic to IGI, excluding those that might result from interactions with the ink support, and to assess how such processes are influenced by ink variability. These processes may be relevant for a better future understanding of the overall deterioration of ink-containing manuscripts, such as ink discolouration or fading, as well as the concomitant color change—from a deep black to a brownish hue—which is typically attributed to the recombination of Fe^2+^ cations (formed via the reduction of Fe^3+^ cations in the complexes, a process that will be investigated in the present paper) with sulfate ions or other organic anions, such as oxalates.^1,3,21,22^ It is noteworthy that Fe^2+^ compounds exhibit higher solubility, which, depending on the conservation conditions, may result in ink migration and, more broadly, enhanced penetration of Fe^2+^ into the substrate, potentially promoting the spread of corrosion-related degradation within the ink-bearing support.^3,21,23^ Finally, it is worth noting that the reduction of Fe^3+^ and the simultaneous oxidation of phenols occur through Fenton and Fenton-like reaction mechanisms (Figure 1).^1,7,24,25^ These reactions also generate reactive radicals, which significantly contribute to the degradation of the ink support.^1,3,11,19,21,26,27^ While an in-depth investigation of manuscripts degradation induced by the presence of IGI would require a dedicated study, the current paper aims to clarify the degradation patterns that may be relevant to consider in such future research.

**Figure 1:**
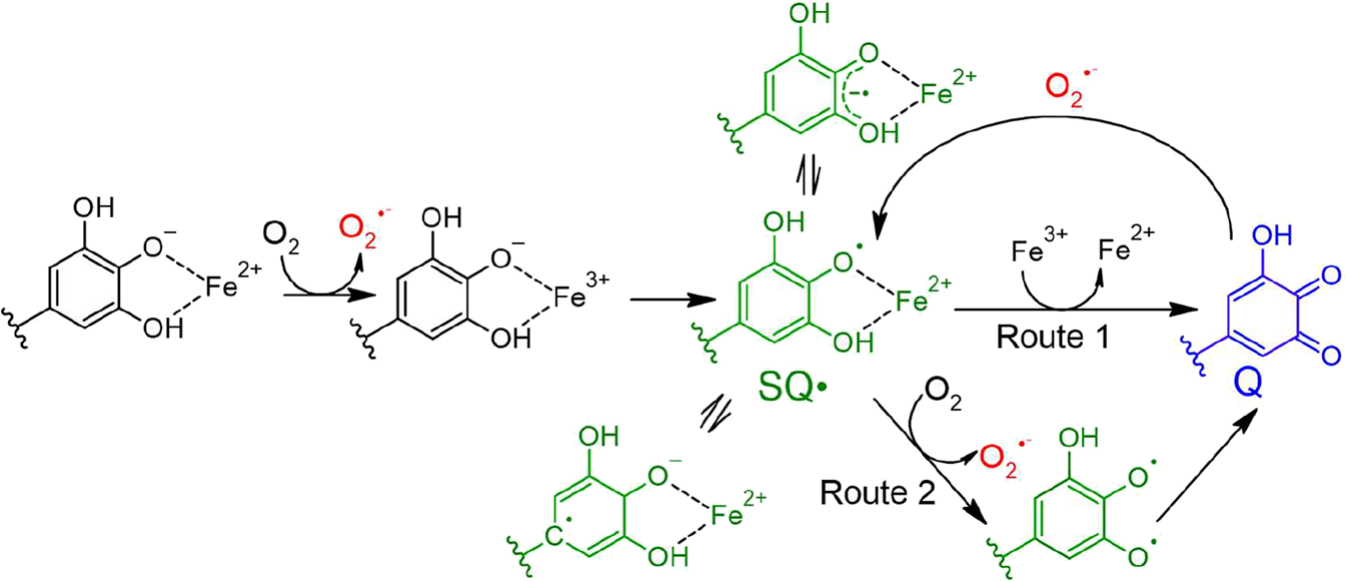
Schematic representation of radical formation mechanisms and pathways involving iron–polyphenol complexes. Superoxide formation is highlighted in red, while the generation of semiquinone radicals (SQ·) and their possible delocalization onto oxygen and carbon atoms are shown in green. The formation of quinone species (Q) is indicated in blue. This scheme serves as a simplified illustration and is not exhaustive, as additional radical-involving processes may also occur.

## 2 Experimental Section

### 2.1 Iron-Polyphenolic Complexes Preparation

Iron complexes of gallic acid (GA) and tannic acid (TA), referred to as Fe-GA and Fe-TA, were synthesized following the method of Caterino et al., in order to investigate different iron-to-ligand stoichiometric ratios across varying pH conditions.^8^ The type of polyphenolic ligand, the iron-to-ligand ratios and pH conditions employed for the preparation of the iron–polyphenolic complexes are consistent with parameters previously employed to investigate the structural variability of such complexes.^8,28^ Since these variables in combination proved critical for structural determination, the same preparation conditions were also applied in the present study to assess how this structural variability affects the degradation patterns.^8^ The experimental design is summarized in Table 1, with triplicate samples prepared for each condition in order to enhance the statistical reliability of the results.

**Table 1:** Summary of the Experimental Design Related to the Study of Reference Iron-Polyphenolic Complexes

type of ligand	pH^a^	Fe:ligand stoichiometry^b^			
GA	2	0.8:1	1:1	1.5:1	5:1
	4	0.8:1	1:1	1.5:1	5:1
	6	0.8:2	1:2	1.5:2	5:2
TA	2	1:1	1.5:1	3:1	5:1
	4	1:1	1.5:1	3:1	5:1
	6	1:2	1.5:2	3:2	5:2

a The pH reported refers to the pH of the ligand buffered solutions prior to the iron salt addition.

b The Fe:ligand stoichiometry has to be intended as ratios of reagents in the complexes’ preparation.

Three CH_3_COOH/CH_3_COONa buffers at pH 2, 4, and 6 were initially prepared using concentrated acetic acid (96%, Merck Sigma-Aldrich) and NaOH solutions, obtained by dissolving NaOH pearls (96%, Merck Sigma-Aldrich) in deionized water.

**Caution!** Glacial acetic acid and sodium hydroxide pearls are classified as GHS Skin Corrosion, Category 1A. To minimize risks of contact and inhalation, both substances were handled with caution, using gloves, goggles, and appropriate ventilation.

To prepare the Fe-GA complexes, concentrated GA solutions (∼12 mg/mL) were made by dissolving GA (97.5–102.5% purity, Sigma-Aldrich) in 100 mL of each buffer. Similarly, the TA solutions (∼100 mg/mL) were prepared by dissolving TA (99% purity, Sigma-Aldrich) in 50 mL of each buffer. Solubilization of GA and TA was achieved using vortex mixing and sonication baths. The final pH was checked with pH indicator strips (Macherey-Nagel). Exact amounts of GA and TA solutions were mixed with freshly prepared ∼250 mg/mL FeSO_4_·7H_2_O solutions (99% purity, Chemsolute Th.Geyer) to achieve the desired iron-to-ligand ratios. The obtained solutions were stored in open Falcon tubes in the dark for at least 10 days to allow the oxidation of Fe­(II)-polyphenol complexes into Fe­(III)-polyphenol complexes.

After oxidation, the Fe­(III)-polyphenol precipitates were separated by centrifugation (14,500 rpm, 15 min), requiring at least three washing cycles. Complete separation was difficult due to the fine size of the particles, leaving some dispersed in the supernatant. The precipitates were then dried in an oven (24 h, 50 °C, 10% ventilation) and ground in an agate mortar. The fine powders were finally pressed into 5 mm diameter pellets with a suitable press die.

### 2.2 OG Extract (Ex) Preparation

Following the procedure described by Caterino et al.,^8^ based on a historical recipe,^1–3^ Aleppo OG extracts (Ex) were prepared as follows. Aleppo oak galls (Kremer Pigmente) were roughly ground then mixed with deionized water at a ratio of 35 mL per gram of sample. The mixture was stirred at room temperature (RT) for 3 days. The insoluble material was removed by Büchner filtration and centrifugation (15 min, 9000 rpm). The clear solution was concentrated using a rotavapor, then freeze-dried to completely remove water. The lyophilized extract was stored in a sealed Falcon tube under Ar at −20 °C to prevent oxidation and hydrolysis.

### 2.3 Fe-Ex Complexes Preparation

Following the protocol described by Caterino et al., two types of Fe-Ex complexes were prepared as follows.^8^ In a first batch, an exact amount of freeze-dried Ex was dissolved in pH 2 buffer, adding small volumes gradually until fully dissolved. In a second batch, an exact amount of freeze-dried Ex was dissolved in water (no buffering), adding small volumes until fully dissolved. The pH of this second solution was measured (Horiba LAQUAtwin) and found to be 3.7 ± 0.2. These solutions were then combined with a freshly prepared FeSO_4_·7H_2_O solution (∼250 mg/mL) to maintain a ratio of 50 mg iron salt per 100 mg extract. The experimental design for this part of the study is summarized in Table 2, with triplicate samples for each condition to improve statistical reliability. The complexes were stored in the dark for 25 days to optimize the yield. Afterward, insoluble particles were isolated by centrifugation and dried under the same conditions used for the Fe-TA and Fe-GA complexes. The fine powders were finally pressed into 5 mm diameter pellets with a suitable press die.

**Table 2:** Summary of the Experimental Design Related to the Fe-Ex Preparation

pH^a^	Ex concentration (mg/mL)	Fe:Ex ratio (w/w)	complex:Ex (in weight)^b^
2 (buffered)	41.76	0.49	0.35 (SD = 0.01)
3.7 (measured)	43.77	0.49	1.53 (SD = 0.02)

a The pH reported refers to the pH of the ligand buffered and unbuffered (Nat. pH = natural pH) solutions prior to the iron salt addition.

b These values represent the Fe-to-ligand ratios for the Fe-Ex complex preparation. The reported ratios are calculated as the averages from triplicate preparations.

### 2.4 Model Inks Preparation

To better represent the composition of real inks, model inks were prepared.^2,5,8^ Following the same procedure reported before, an Aleppo OG extract (Ex_S) has been prepared. 5.7 g of roughly ground Aleppo OG have been mixed with 200 mL of deionized water and stirred at RT for 3 days. Insoluble material was removed by Büchner filtration and centrifugation (15 min, 9000 rpm). The clear solution was then concentrated using a rotavapor as to reduce the final volume up to 50 mL.^2,5,8^

Natural AG (Kremer Pigmente) was purified by dissolving it in deionized water under moderate heating (∼60 °C). The concentrated solution was centrifuged (10,000 rpm, 10 min) to remove insoluble woody residues. The clear supernatant was then poured into silicone molds and left to solidify in an oven under gentle heating (∼72 h, 50 °C, 10% ventilation).

As to have reliable and representative model inks, three batches of inks having different proportions of oak gall extract (here expressed in terms of initial oak gall mass), iron sulfate and AG have been prepared. The experimental design for this part of the study is summarized in Table 3, with triplicate samples for each condition to improve statistical reliability.

**Table 3:** Summary of the Experimental Design Related to the Model Inks Preparation

Fe:OG:AG^a^	mL of Ex_S^b^	mL of FeSO_4_·7H_2_O 250 mg/mL	g of AG
1:1: 1	10	4.56	1.14
1:1.5:1	10	6.84	1.14
1.5:1: 1	10	3.04	0.76

a The ratios defined as Fe:OG:AG represent the mass proportions of the three ink components, Fe therefore standing for mass (g) of iron salt, OG as mass (g) of oak galls and AG mass (g) of Arabic gum.

b Ex_S refers to the Aleppo oak gall extract prepared as previously described, using 5.7 g of initial oak galls in a final volume of 50 mL.

The model inks have been stored in open Falcon tubes in the dark for 10 days to allow oxidation. Subsequently, a thick layer of ink has been deposited on clean glass slides and left to dry (∼72 h). Once completely dried, the solidified ink layer has been scratched off and ground in an agate mortar. The resulting fine powders have been finally pressed into 5 mm diameter pellets with a suitable press die.

### 2.5 UV Aging

In order to simulate an indoor UV aging, the pellets have been aged in a UVA CUBE 400 light chamber (Honle Group, Germany), operating with a halogenide high-pressure lamp (SOL-500). A UVB cutoff glass filter (H1 filter, Honle Group, Germany) has been introduced to better simulate the indoor type of light exposure (long-pass filter with a cutoff at 315 nm). The intensity of UVA and UVB radiation was measured with a UV-Meter (Honle Group, Germany), operating specially designed sensors across the entire internal surface of the chamber. The average irradiation inside the chamber was 5.1 ± 0.1 mW/cm^2^ for the UVA radiation (315–400 nm) and 0.8 mW/cm^2^ for UVB radiation (280–315 nm). The chamber temperature, which cannot be controlled, was regularly monitored and reached a maximum of around 45 °C.

Based on previous studies, UV aging was conducted by irradiating the samples for a total of 750 h, divided into five distinct aging phases, here defined as follows: UV1 = 12 h, UV2 = 80 h, UV3 = 250 h, UV4 = 390 h, and UV5 = 750 h.^29^ The reported hours represent cumulative irradiation time. At the end of each aging phase, the samples were allowed to cool to RT for few minutes before being characterized. They were then placed back into the light chamber to start the following aging phase.

### 2.6 Relative Humidity (RH) Aging

RH aging was performed using a custom-built system consisting of a gas mixing unit connected to a chamber designed for gas aging. A synthetic air stream at approximately 1 bar (Messer, Germany) is divided into two flows, one of which passes through a humidifier containing Milli-Q water. The two streams are then recombined in the mixing unit before entering the chamber. The mixing ratio of the two streams was manually adjusted via the flowmeters (InFlux, England) to achieve the selected RH levels for this study: 30% and 80% respectively, simulating a dry and humid indoor environment. The RH level in flux was measured using a suitable sensor (ELV, Germany) before connecting it to the chamber. The samples were exposed to the constant humidified flow for a total of 168 h. The Fe-GA, Fe-TA and Fe-Ex sets of samples were aged with both 30 and 80% RH, while the model inks were aged just with 80% RH.

### 2.7 Raman Characterization

Raman characterization was conducted using a portable ProRaman-L-Dual-G instrument (Enwave Optronics), equipped with a Leica Microsystem long working distance (LWD) 50× objective lens (connected to a digital camera) and a CCD detector with an autorefrigeration system maintaining a constant detector temperature of −60 °C. For each pellet, three Raman spectra were collected using a 785 nm laser (instrumental resolution of 7 cm^–1^). The acquisition parameters for the entire study were set consistently with those previously published by Caterino et al.: three scans of accumulation, 15 s of acquisition time, and a laser power ranging from 0.7 to 1.7 mW (measured subsequently with a ThorLabs 350-1 power meter, Germany). The spectra were processed through baseline correction, averaging, normalization (using the min–max method), and smoothing using OPUS, and were analyzed in detail with Origin2018.^8^ For spectral analysis, the local maximum algorithm of Origin2018 peak analyzer (local maxima algorithm) was used to automatically and consistently determine peak positions and heights. Raman-derived parameters and spectra were then obtained by first averaging the data per sample (pellet) and subsequently averaging the triplicate sample means.

### 2.8 IR Characterization

The characterization via Fourier Transform Infrared spectroscopy (FTIR) has been carried out using the Bruker Alpha-P instrument in Attenuated Total Reflection (ATR) mode. The ATR module has a fixed diamond crystal with a dimension of 2 × 2 mm^2^, upon which the sample pellets have been placed and gently pressed. Considering the dimension of the crystal, a single spectrum (4000–400 cm^–1^) has been acquired for each pellet using 64 scans of accumulation with a resolution of 4 cm^–1^. The spectra have been finally processed (baseline correction, averaging, normalization, and smoothing) using OPUS, and analyzed in depth using Origin2018.

### 2.9 Electron Paramagnetic Resonance (EPR) Spectroscopy Characterization

The EPR characterization has been carried out in Continuous Wave (CW) mode at RT using the Elexsys E 500 instrument by Bruker. The instrument is designed for measurements in the X-band (frequency of the radiation of 9.75 GHz) equipped with a high sensitivity cavity SHQE1119 by Bruker Biospin GmbH. For all the measurements, approximately half of an aged pellet has been ground and the resulting fine powder (in the order of few milligrams) has been placed in small capillaries (1.15 ± 0.5 mm diameter micro hematocrit tubes by BRAND), properly sealed with Critoseal. The capillaries have then been placed into clean EPR tubes to be analyzed. The parameters for signal acquisition were configured as follows: microwave power was set to 20 mW, with a power attenuation of 10 dB. The modulation amplitude was adjusted to 8 G, and the modulation frequency was set at 100 kHz. The magnetic field was centered at 3500 G, with a total field sweep of 6000 G. The number of acquired scans per measurement ranged from 3 to 5, with an acquisition time for each scan of 90 s (conversion time was 87.89 ms, RC time constant automatically set to 1/4 of the conversion time). The adjustment of the instrumental parameters for the tuning was automatically performed by the instrument through the Xepr Software (Bruker). The spectra have been finally processed and analyzed using Origin2018. No baseline correction was applied. A normalization of the signal intensities was carried out based on the samples’ weight, along with field corrections accounting for the magnetic field at which each sample was tuned. The signal line width was estimated through the peak–peak distance calculated using the Origin2018 software.

## 3 Results and Discussion

### 3.1 Overview of Degradation-Related Spectral Changes

The spectroscopic indicators obtained from the acquired data are introduced here as the basis for the forthcoming comparison aimed at elucidating the impact of each variable on degradation.

Raman spectroscopic data turned out to be among the most effective in the monitoring of the aging processes occurring in the whole set of samples involved in the study. All the acquired spectra are dominated by three main bands: two very strong signals which can be encountered in the spectral ranges 1310–1350 and 1465–1485 cm^–1^, and a medium-strong broad band in the range of 450–650 cm^–1^, denoted here respectively as A, B and F band in accordance with Caterino et al. (Figure 2B).^8^ Previous studies have clarified that the A band, which can in principle be assigned to C–O stretching vibrations (ν­(C–O)) of phenolic C–OH groups coupled with C–H bending (δ­(C–H)) and aromatic stretching vibrations, along with the B band, receives its main contribution from C–O stretching vibrations of those phenolic groups involved in the complex formation.^8,30–35^ The B band instead seems to receive a larger contribution from ring vibrations, specifically the benzene *19b* vibration, which is coupled with ν­(C–O) and δ­(C–H) motions. It has been previously demonstrated that the position of these two strong signals, and in particular the one of the A band, is strongly dependent on the type of polyphenolic ligand interacting with iron cations, as to say the chemical environment of the phenolic groups involved in the complexes formation.^8,31,36^

**Figure 2:**
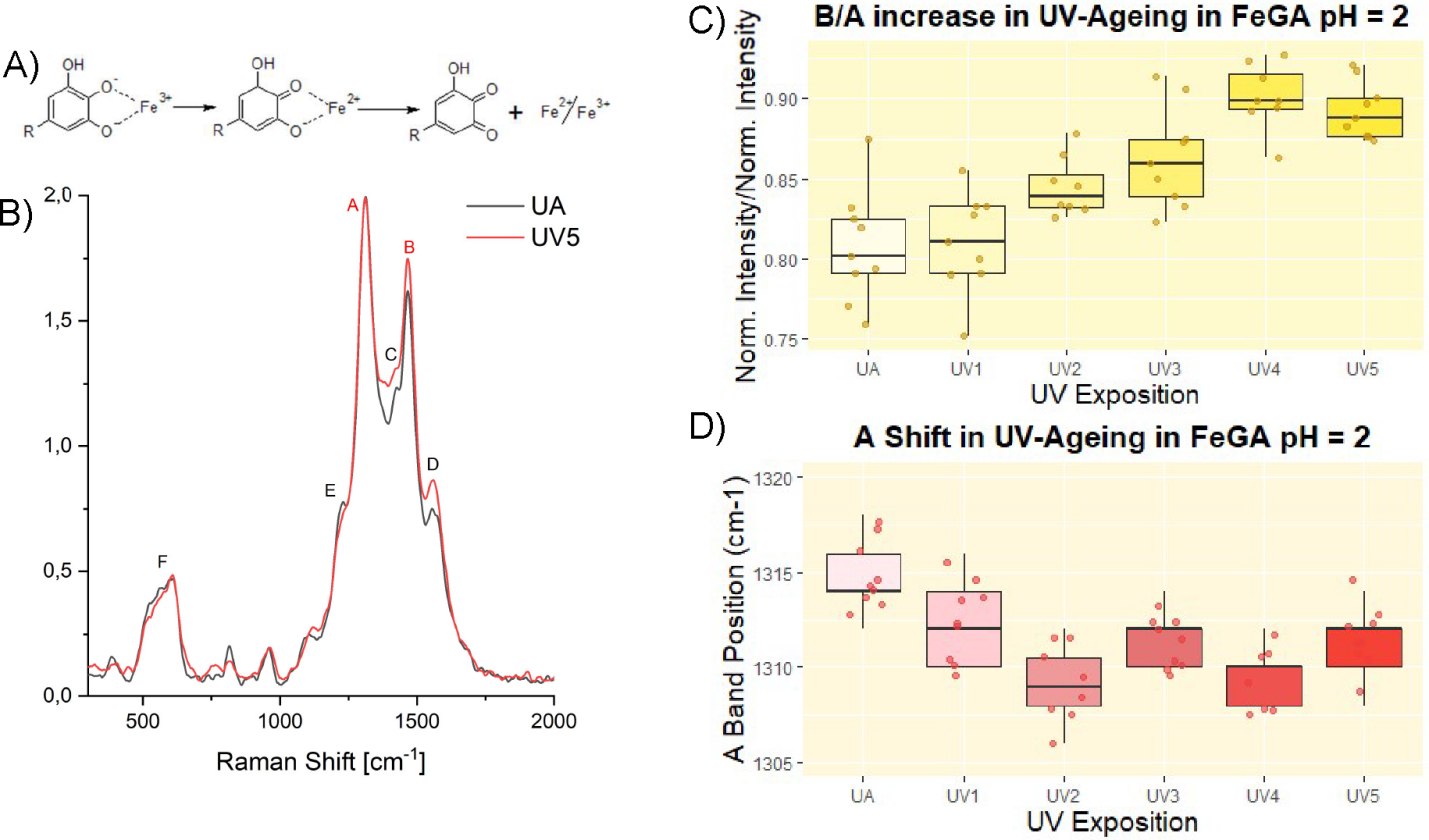
(A) Simplified mechanism of the typical oxidation reaction leading to the complexes’ degradation. The oxidation process follows a typical Fenton radicalic pathway, not reported here in detail, which can be considered autocatalytic (the reduced ferrous cations can be oxidized back to the ferric form). (B) Raman spectra of FeGA complexes prepared at pH = 2 with the iron-to-ligand ratio 0.8:1. For each of the three independently prepared samples (triplicates), three spectra were acquired. The reported spectra represent the average of the three per-sample averages and highlight some of the most noticeable differences associated with the UV-aged samples. (C) Boxplots showing the increasing trend of B/A during the UV aging. (D) Boxplots showing the displacement of the A band, not clearly visible simply looking at the spectra, during the UV aging. Both the boxplots (C, D) are related to the aging results of the subset FeGA at pH = 2 prepared with the iron-to-ligand ratio 0.8:1. In the boxplots reported, the data points are also shown as to better visualize the data distribution. Outliers were excluded using the 1.5 × interquartile (IQ) rule (values below 1.5 × 1st IQ and above 1.5 × 3rd IQ). UA: unaged.

Based on the evidence reported by Caterino et al.,^8^ which is also in good agreement with the previous results of Espina et al.,^31^ it has been demonstrated, by combining Raman and EPR data, that the relative intensity of the A band can provide a rough estimate of the so-called “metal–polyphenolic network density,” as to say, the degree of cross-linking between metal and polyphenolic ligands within the complex. In the adopted spectral processing method, after min–max normalization the A band remains the most intense feature in most spectra (Figure 2A). For this reason, its variation in intensity is represented by the B/A parameter, defined as the ratio of normalized peak heights, which avoids the need for deconvolution.

In all the sets of samples involved in the current study, this parameter visibly increases during the accelerated UV aging process. The increase of this parameter, in good agreement with previous reported data,^37^ can therefore be associated with a reduction in the “metal-polyphenolic network density”, as to say a loss of phenolic-metal interactions, likely due to the conversion of the phenolic moieties into semiquinonic or quinonic ones through oxidation (Figure 2A–C).^1,2^

Along with the increase of B/A, shifts in the position of the A band have been observed during the UV aging. The A-band shift toward slightly lower wavenumbers, with median shifts of up to ∼10 cm^–1^, likely reflects the loss of water from the solid complex particles, which reduces structural order and increases the amorphous character of the complexes (although the instrument resolution is 7 cm^–1^, the observed shifts generally exceed the standard deviation of the A-band position distributions, suggesting that these changes are meaningful, even if they cannot be interpreted as exact values).^37–39^ This phenomenon is therefore associated with a change in the coordination sphere of iron, but it cannot be directly associated with degradation patterns, such as oxidation. This process is likely to be reversible: in many of the tested sample sets, the distribution of A-band positions appears to shift toward slightly higher wavenumbers following RH aging (Figure 3A). On the contrary, the position of the B band, appears more stable, and no major shifts have been observed during accelerated aging.

**Figure 3:**
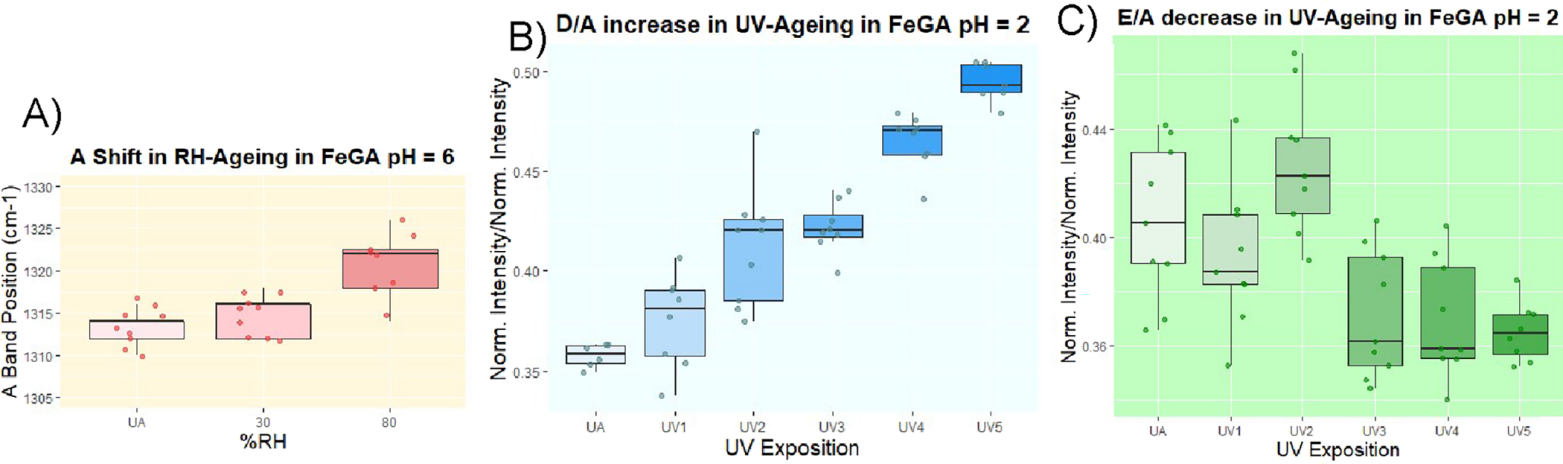
(A) Boxplots illustrating the shift of the A band toward higher wavenumbers during RH aging, attributed to changes in the coordination environment of iron that affect C–O stretching vibrations (UA: unaged). The data correspond to the FeGA subset at pH 6, prepared with an iron-to-ligand ratio of 1:2. (B) Boxplots displaying an increasing trend in the D/A parameter during UV aging. (C) Boxplots showing a decreasing trend in the E/A parameter during UV aging. The boxplots in panels B and C pertain to the FeGA subset at pH 2, prepared with an iron-to-ligand ratio of 0.8:1. In all cases, outliers were excluded using the 1.5 × IQR criterion.

The bands A and B are not the only ones affected by the degradation processes. For instance, the intensity signal lying in the range 1560–1580 cm^–1^, here defined as D, follows a similar trend to that of the B band: since the D band, as the B one, is associated with ring vibrations, the relative intensity D/A appears to increase during the UV-aging due to the reduction in intensity of the A band (Figure 3B). It is worth noting that, unlike the intense B signal, the D band in spectra from real samples is often masked or partially overlapped by other signals, making its intensity and position difficult to determine reliably.^8,31,35^ Therefore, the use of the B/A parameter instead of D/A to infer the degradation state of IGI in real samples is more suitable and more likely to yield accurate estimations.

The intensity of the signal envelope in the 1220–1240 cm^–1^ range, here referred to as E and primarily associated with C–O stretching vibrations, appears to be influenced by several factors beyond the decrease of the A band.^8,31,34^ As a result, while the B/A and D/A parameters exhibit more consistent trends across the different data sets during aging, the E/A parameter proves to be more variable and challenging to interpret (Figure 3C). The signals related to Fe–O interactions, located in the 450–650 cm^–1^ region, may also undergo modifications (see Supporting Information Figure F.S1). It is worth noting that the broad band visible in this area, here referred to as F, actually represents the envelope of several overlapping Fe–O vibration modes.^8,31,38^ Considering the intrinsic complexity of this spectral region and the challenges in accurately following the behavior of these signals across all subsets, band F cannot be reliably used to monitor aging processes.

The modifications induced by the artificial aging can also be investigated through the ATR-FTIR spectroscopic data. The most interesting spectral area to evaluate iron-polyphenolic structural changes in artificial aging is the range 1800 – 400 cm^–1^. As previously reported,^8,28,30^ the FTIR spectra in this region show numerous signals whose intensity and position strongly depend on the polyphenolic ligand, pH, and Fe concentration. Given this complexity, aging effects are best interpreted by examining spectral variations on a subset-by-subset basis. Excluding patterns specific to isolated subsets, common trends can be for instance identified across the whole FeGA set. UV-induced oxidation of phenolic moieties causes a strong decrease in the intense bands at ∼1200 cm^–1^ and ∼1065–1080 cm^–1^, which are typically associated with ν­(C–O) vibrations coupled with δ­(C–H) and δ­(C–OH). The medium-intensity band at ∼1240 cm^–1^, likely related to similar type of vibrations, gradually disappears (Figure 4).^5,8,28,30,31^ Significant changes are also observed in the 1750–1300 cm^–1^ region. The 1750–1600 cm^–1^ range shows a broad envelope of carbonyl and aromatic C=C signals, often coupled with δ­(C–H), and is strongly affected by water O–H bending, complicating interpretation. In the 1450–1300 cm^–1^ region, the band at ∼1310–1320 cm^–1^, arising from ν­(C–O) vibrations, and the peak at ∼1380 cm^–1^, corresponding to carboxylate C=O stretching, together provide an indication of structural rearrangements in the complexes.^5,28,30,31^

**Figure 4:**
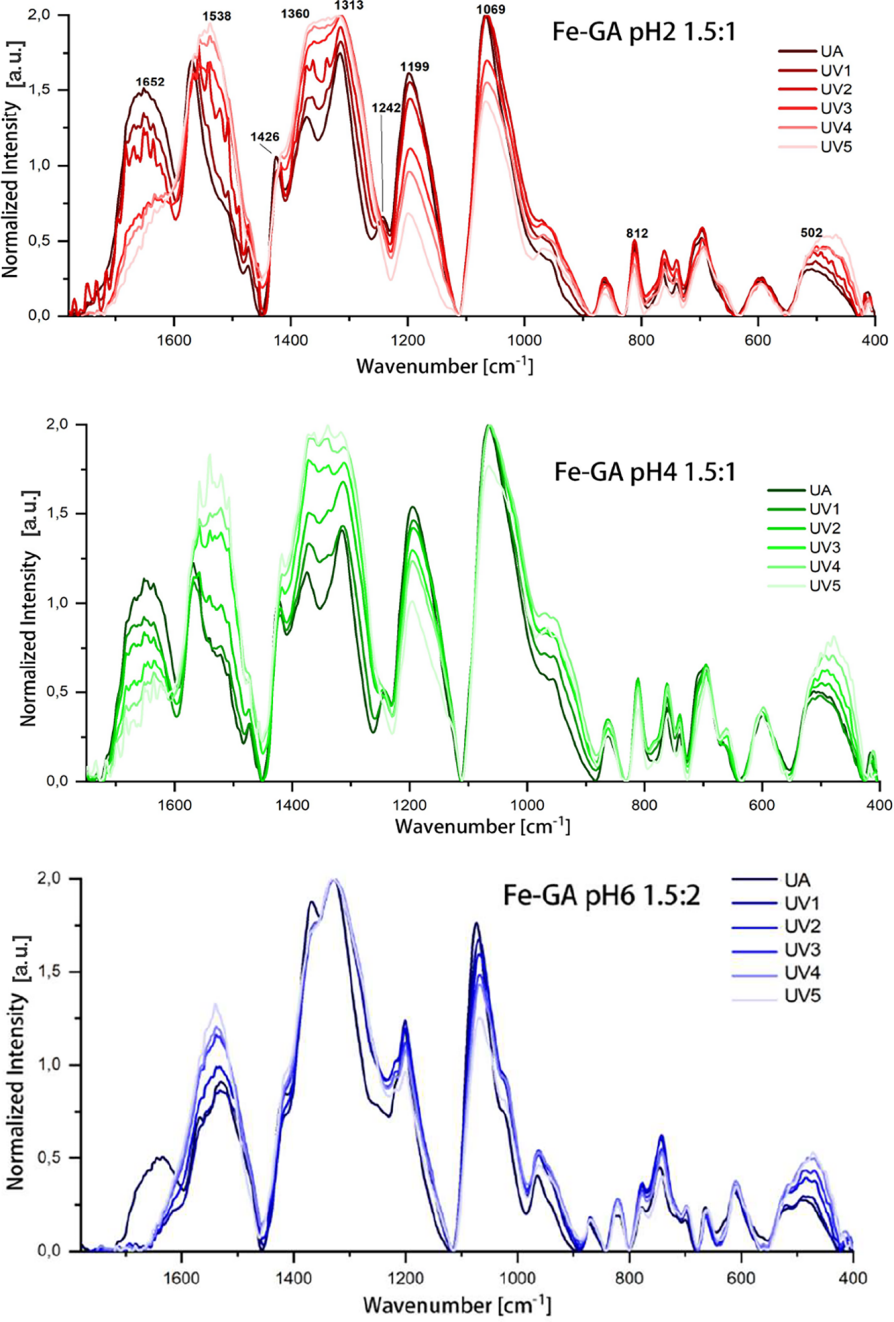
ATR-FTIR spectra of Fe-GA complexes during UV aging: (A) Fe-GA pH 2, 1.5:1; (B) Fe-GA pH 4, 1.5:1; and (C) Fe-GA pH 6, 1.5:2. Each spectrum represents the average of measurements acquired on triplicate pellet samples.

However, these general trends observed in FeGA have not been observed in RH aging. Moreover, FeTA, Fe-Ex and model inks showed as well completely different patterns. The variations observed in these samples are in fact less pronounced than those seen in the FeGA samples. This suggests that, in complexes containing larger polyphenols as organic ligands (as expected in real IGI), the changes induced by artificial aging produce modifications that are more difficult to detect by FTIR. Variations in the general spectral profile and main bands intensity do not follow a specific trend: variations in the intensities of the signals and just in few cases significant shifts are mainly observed in the 1750–900 region with more pronounced changes in the sub-region 1750–1300 and 1150–900 subregion in FeTA. Excluding specific patterns that will be later discussed, Fe-Ex and model inks instead did not show particularly pronounced changes in the FTIR spectra (Figure F.S2).

The overall change in the iron coordination environment, resulting from the weakening of metal–phenolic interactions during the oxidation process, can also be supported by CW-EPR spectroscopy. As reported in the literature, the spectra are characterized by a prominent signal at *g* = 2, attributed to Fe^3+^ ions in their typical octahedral coordination geometry, along with a minor signal around *g* = 4.3, associated with tetrahedral coordination.^8,40,41^ The latter signal is generally attributed to glass impurities from the capillaries used in the analysis, with the exception of the Fe-TA complex subset prepared at pH 6 (see also the Supporting InformationFigure F.S3).^8^ The increased heterogeneity in the Fe^3+^ coordination environment and the weakening of coordination bonds due to oxidation are reflected in the broadening of the *g* = 2 signal, as shown in Figure 5A,B (see also Supporting Information Figure F.S4).

**Figure 5:**
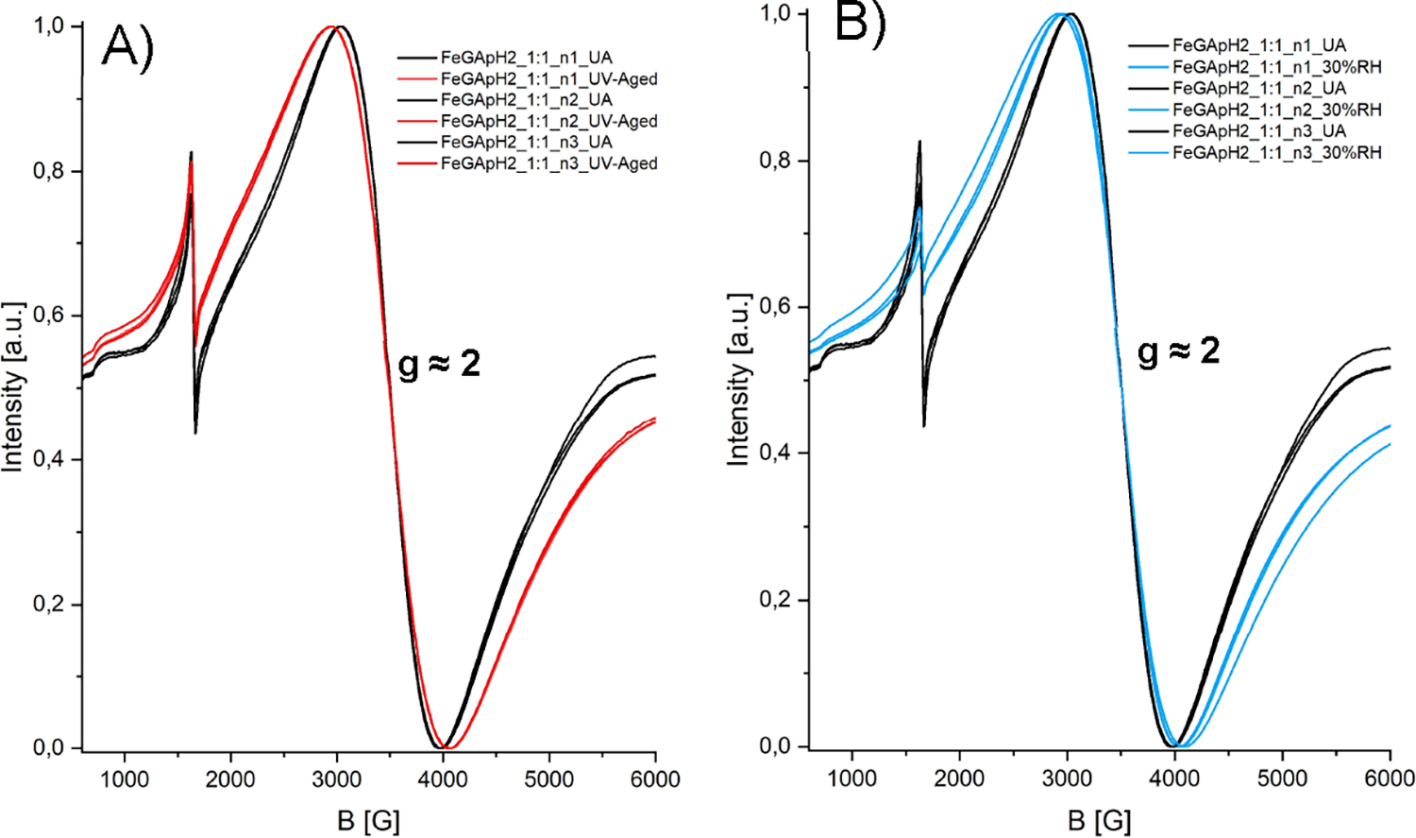
CW-EPR spectra of the Fe-GA subset at pH 2 with an Fe:GA ratio of 1:1. (A) Spectra recorded before and after UV aging. (B) Spectra recorded before and after aging at 30% RH. In both cases, aging leads to a noticeable broadening of the *g* = 2 signal, indicating changes in the coordination environment.

Due to the slower relaxation time of the Fe^3+^ paramagnetic species, just in very few cases it has been possible to directly observe the formation of organic radicals (Figure 6). The g factor observed in these systems (*g* ≈ 2.00276) is coherent with the presence of radical delocalized on carbons of aromatic structures.^42,43^ These isolated observations do not allow to have a proper statistics to better investigate the phenomenon of radical formation, which can be instead better understood throughout the use of spin-traps, as also recently demonstrated by Teixeira et al.^44,45^

**Figure 6:**
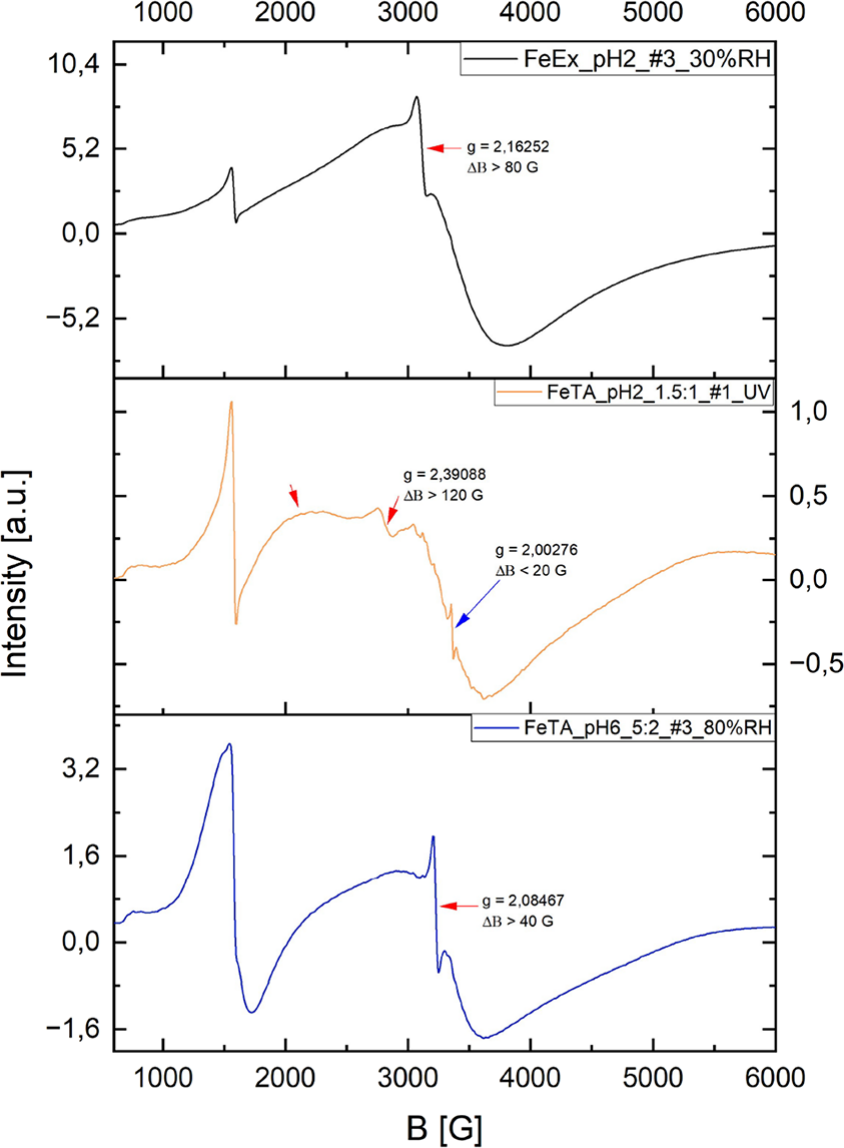
CW-EPR spectra showing signals attributable to SPIONs and/or organic radicals. Red arrows indicate features likely associated with SPIONs of superparamagnetic dimensions, while the central panel highlights a radical signal marked by a blue arrow. The calculated *g*-values and corresponding line widths (Δ*B*) are also reported.

Similarly, sporadic signals consistent with the formation of superparamagnetic iron oxide nanoparticles (SPIONs) were observed throughout the CW-EPR measurements, in agreement with previous reports (Figure 5).^8,46^ SPION-related signals are typically characterized by broad line widths in EPR spectra recorded at room temperature.^47^ However, as demonstrated by Noginova et al., the line width and resonance position are highly dependent on the nanoparticle size. The present results (both in terms of line width and resonance field) suggest the formation of SPIONs with variable dimensions, including very peculiar SPION systems within the 5–30 nm range which display an antiferromagnetic behavior (see also Figure F.S5 in the Supporting Information).^47,48^

Having defined the main observable changes in the spectral data associated with degradation phenomena, particularly oxidation, it is now possible to explore the role of the variables considered in this study, namely, the pH prior to Fe addition, the Fe concentration, the structure of the polyphenolic ligands, and the type of aging (UV radiation and RH) in determining the extent and kinetics of the degradation pattern.

### 3.2 Type of Accelerated Aging

A comparison of the data sets corresponding to the two types of accelerated aging protocols (UV exposure for up to 750 h and exposure to 30 and 80% RH constant flux for 168 h) reveals notable differences. In most cases, UV aging results in a more pronounced and clearly detectable oxidation of the complex compared to both RH aging protocols. This is evidenced by a more substantial increase in the Raman parameters B/A and D/A, as previously discussed, which in the case of RH aging do not always exhibit significant variations (Figure 7, see also Supporting Information Figure F.S6).

**Figure 7:**
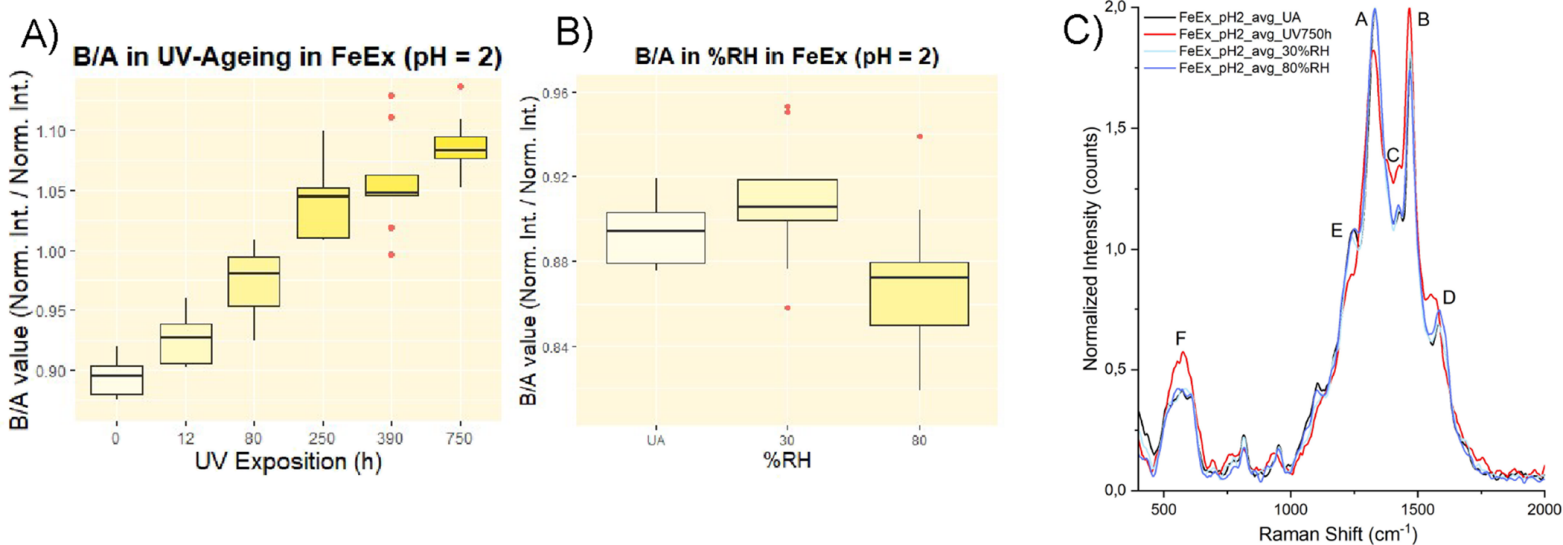
(A) Boxplot showing the increase in the B/A Raman-derived parameter after UV aging. (B) Boxplot comparing B/A values in unaged (UA) and humidity-aged samples. Both plots refer to the Fe-Ex subset at pH 2. Outliers (in red) were excluded from statistical analysis using the 1.5 × IQR criterion. (C) Overlapped average Raman spectra of unaged, UV-aged, and humidity-aged samples (9 spectra per condition: 3 per pellet × 3 pellets).

As briefly noted in the previous paragraph, another key difference between the two aging protocols pertains to the changes potentially associated with the water content in the solid particles of the iron–polyphenolic complexes. The reduction in water content during UV aging not only affects the position of the Raman A band (resulting in a shift toward slightly lower wavenumbers, see Figure 2D), but is also reflected in several spectral changes observed in the IR analyses. For instance, in the spectral range 1715–1530 cm^–1^ of the spectra related to Fe-GA complexes, two prominent bands are detected (Figure 8A): a broad band centered around 1650 cm^–1^, attributed to vibrations of free carboxylic moieties, and a band at approximately 1570 cm^–1^, associated with vibrations of COO^–^ groups bound within the complex.^5,8,30,31^ Across all Fe-GA subsets, UV aging induces a marked decrease in the intensity of the first band and a concomitant increase and broadening of the second. These spectral profile changes may be interpreted as a consequence of water loss, which promotes deprotonation of free carboxyl groups and their subsequent complexation to Fe^3+^ ions, thereby contributing to complex formation. This interpretation is further supported by the fact that the decrease in the band centered around 1650 cm^–1^ is never detected under RH aging conditions, and on the contrary, in most cases, a slight increase in this band is observed under these conditions (Figure 8B).

**Figure 8:**
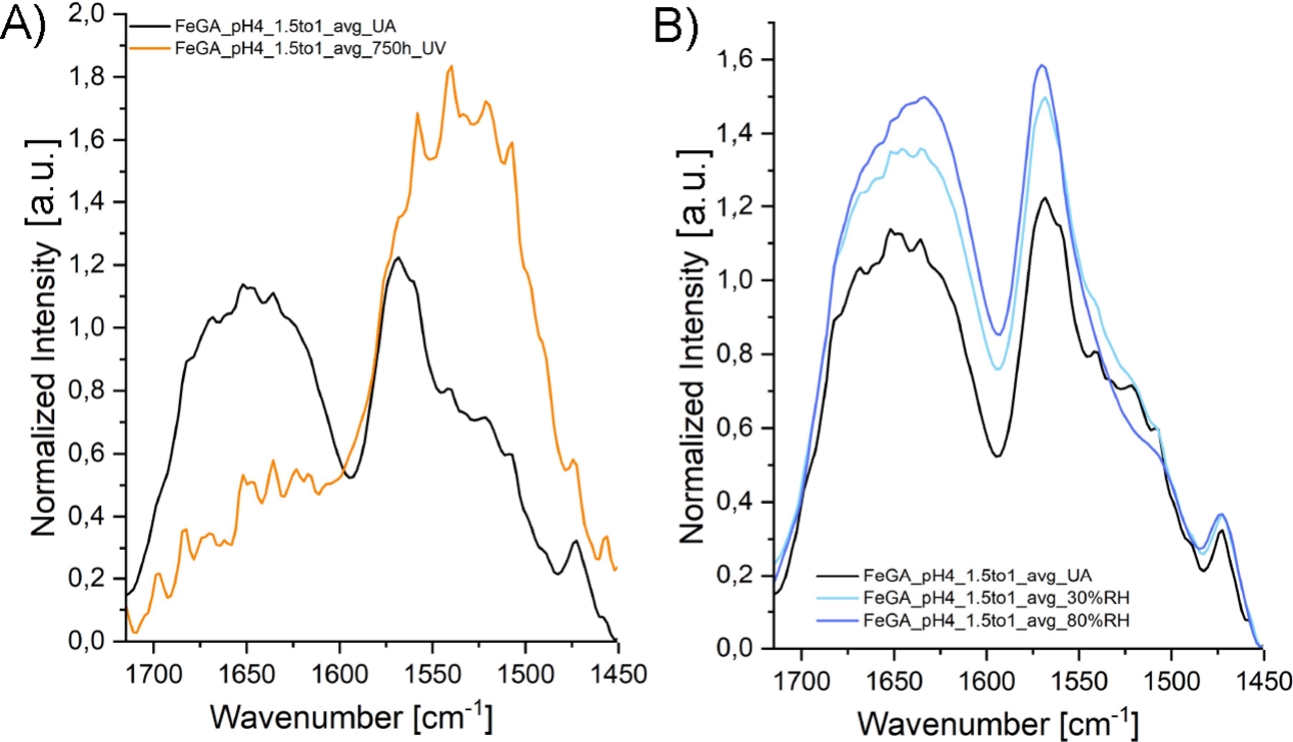
Average FTIR spectra for the Fe-GA subset pH 4, Fe:GA = 1.5:1. (A) Spectra of unaged (UA) and UV-aged samples. (B) Spectra of UA and RH-aged samples. In both cases, the spectra represent the average of three measurements (one per pellet) and are displayed for clarity within the 1730–1450 cm^–1^ range.

As expected, in the majority of the cases, exposure to 30% RH did not produce a significant effect on the aging of the samples. For this reason, for the model inks, the artificial aging was conducted exclusively under 80% RH conditions.

The interpretation of EPR data is more complex, as it is influenced by multiple factors. Nevertheless, with some exceptions that will be discussed later, it can be stated that UV aging generally leads to a higher increase in line width of the *g* ≈ 2 signal compared to that observed under RH aging (Figure 9) This observation is consistent with the other spectroscopic data presented, suggesting that UV exposure more effectively promotes degradation processes, resulting in a more heterogeneous distribution of coordination centers, namely facilitating the complexation of Fe^3+^ ions at less specific sites.

**Figure 9:**
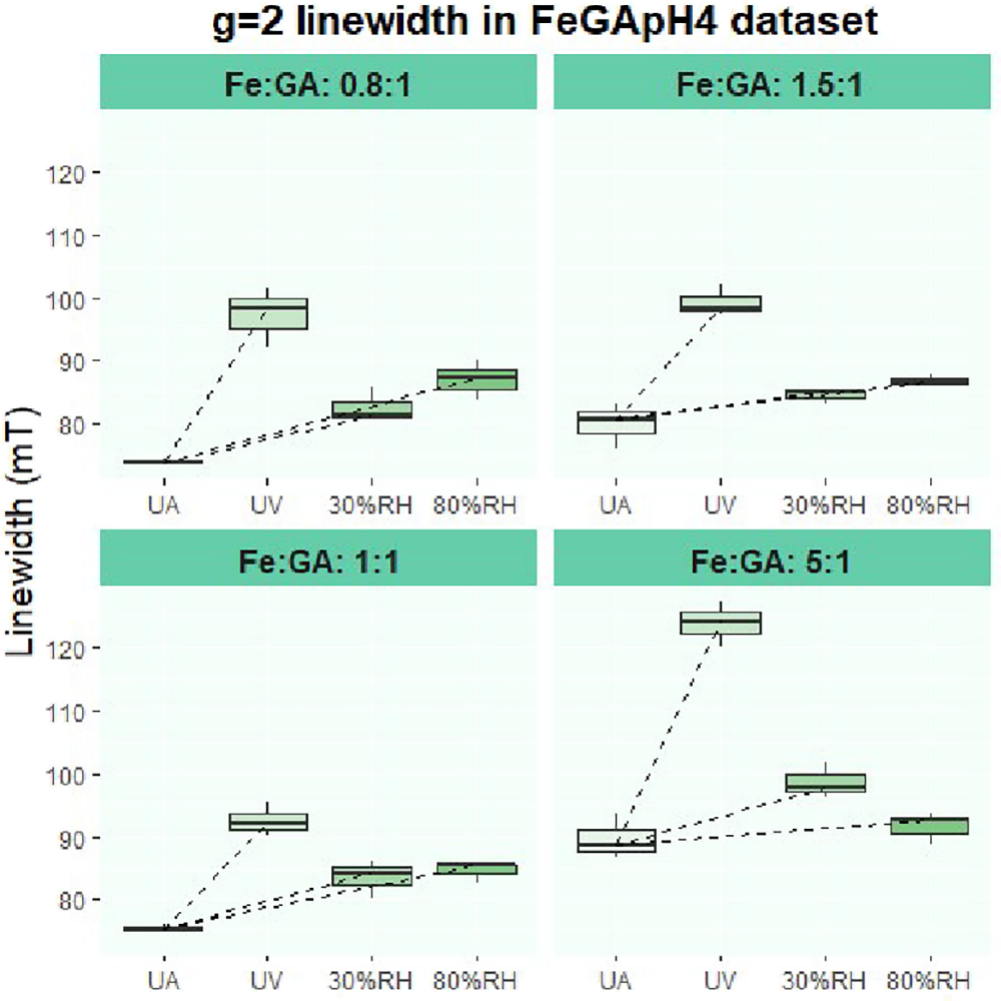
Boxplots showing the increase in EPR signal line width at *g* ≈ 2 due to aging in the Fe-GA set pH 4 (the data for all Fe:GA subsets are presented to highlight the common trend observed across the different iron-to-GA ratios within this set). Each boxplot represents the distribution of three measurements (one per pellet).

Interpreting EPR results in the context of aging is particularly challenging due to the simultaneous occurrence of multiple processes. Also in this case, beyond ligand oxidation, variations in water content play a crucial role, as they can significantly modify the coordination environment and, in turn, the resulting EPR spectra. Further contributing factors, discussed in detail below, add to the complexity of interpretation.

### 3.3 Role of the Type of Polyphenolic Ligand

A comparison of the data acquired on unaged and aged iron complexes prepared with different ligands and ligand sources reveals that degradation processes in Fe-GA are, in general, more homogeneous across the various pH values and Fe-to-ligand ratios studied, compared to the other sample sets. In Fe-GA, as previously mentioned, an increase in the B/A Raman-derived parameter is observed, indicating a ligand oxidation (see also Supporting Information Figure F.S7). In some subsets, this is accompanied by slight shifts in the distribution of A-band positions, attributed to a reduction in water content, with the extent of these changes varying as a function of pH and iron concentration. In contrast, for complexes with larger ligands, such as those in Fe-Ex, the model inks, and TA, the overall trends differ substantially across subsets, suggesting more diverse iron coordination environments and more heterogeneous degradation patterns.

While an increase in the B/A ratio is consistently observed across the different pH and Fe conditions, though with varying magnitudes, a shift of the A band toward higher wavenumbers (up to 12 cm^–1^) is detected in some subsets of Fe-TA samples. This effect is particularly evident in Fe-TA pH 2 during UV and RH aging (Figure 10A, see also Supporting Information Figure F.S8), but is also visible to a lesser extent in other Fe-TA subsets, such as Fe-TA pH 4 during RH aging (see also Supporting Information Figure F.S8 B). Interestingly, this specific trend correlates with the EPR data, where the same samples exhibit a decrease—rather than the typical increase—of the line width in the *g* ≈ 2 region upon aging (Figure 10B, see also Supporting Information Figure F.S9). This correlation may reflect the dual effect that changes in water content can have on the iron coordination environment. On one hand, dehydration may weaken the phenolic–iron coordination (i.e., induce structural relaxation), leading to a red shift of the Raman A band and an increase in the EPR line width. On the other hand, dehydration may also disrupt hydrogen bonding and reinforce metal–polyphenol interactions, resulting in a blue shift of the Raman A band and a decrease in the EPR line width. The predominance of one mechanism over the other appears to depend on both pH and iron-to-ligand ratio. In the first scenario, the water loss responsible for structural relaxation is likely associated with non-coordinated water residing in the so-called “channels” of the iron–polyphenol solid complexes.^38,49^ In the second scenario, where water loss leads to strengthened metal–polyphenol interactions, the water involved is likely that which is directly coordinated to Fe^3+^.

**Figure 10:**
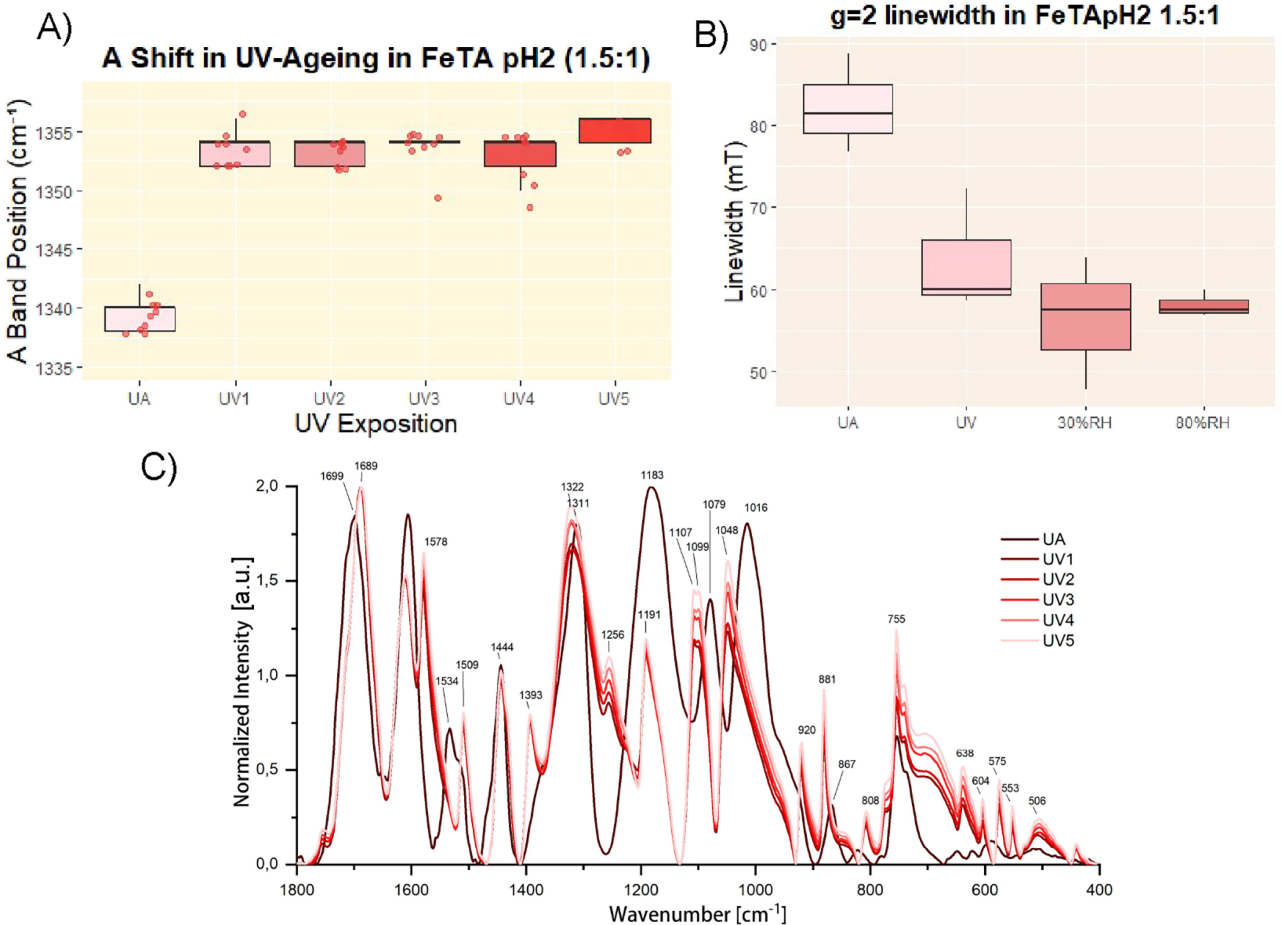
(A) Boxplot showing the pronounced blue shift of the Raman A band observed during UV aging in the Fe-TA subset pH 2, Fe:TA = 1.5:1. (B) Boxplot illustrating the decrease in EPR signal line width at *g* ≈ 2 due to aging in the same subset (data for unaged, UV-aged, and RH-aged samples are shown). (C) Averaged FTIR spectra corresponding to UV aging of the same subset.

An alternative hypothesis is that, in these samples, UV and RH aging trigger hydrolytic processes—namely, the acid-catalyzed cleavage of depside and/or sugar–gallic acid ester bonds which alters the structure of polyphenolic ligands and increases the availability of phenolic −OH groups (see also Supporting Information Figure F.S10).^50–52^ These groups may then coordinate more effectively with Fe^3+^ ions, strengthening the iron–polyphenol interactions. This process could account for the observed blue shift of the Raman A band and the narrowing of the CW-EPR line width at *g* ≈ 2. This hypothesis may also explain the distinctive FTIR features observed in specific Fe-TA subsets, particularly Fe-TA pH2. While in most Fe-TA samples the FTIR changes induced by UV aging are limited to variations in relative band intensities, with only occasional emergence of new features, Fe-TA pH2 exhibits a more substantial transformation in the spectral profile (Figures 10C and 11B, see also Supporting Information Figure F.S11). Notably, new bands appear at approximately 1395 and 1255 cm^–1^, accompanied by a marked decrease in intensity of the ∼1185 cm^–1^ band. Additionally, blue shifts are observed for the bands initially centered at ∼1015, ∼1080, and ∼1534 cm^–1^, which shift to ∼1050, ∼1110, and ∼1570 cm^–1^, respectively. These shifts may be related to structural modifications affecting, in order, the C–O stretching vibrations, the δ­(CH) and ring stretching modes, and the δ­(C–OH) coupled with ring stretching and δ­(CH) vibrations.^5,8,30,31^ These alterations, along with broader changes in the 890–400 cm^–1^ region, are consistent with the occurrence of ligand hydrolysis (Table 4). As might be noticed from Figure 10C, the hydrolytic process seems to start within the first phases of the aging (within the first 12 h of UV exposure).

**Figure 11:**
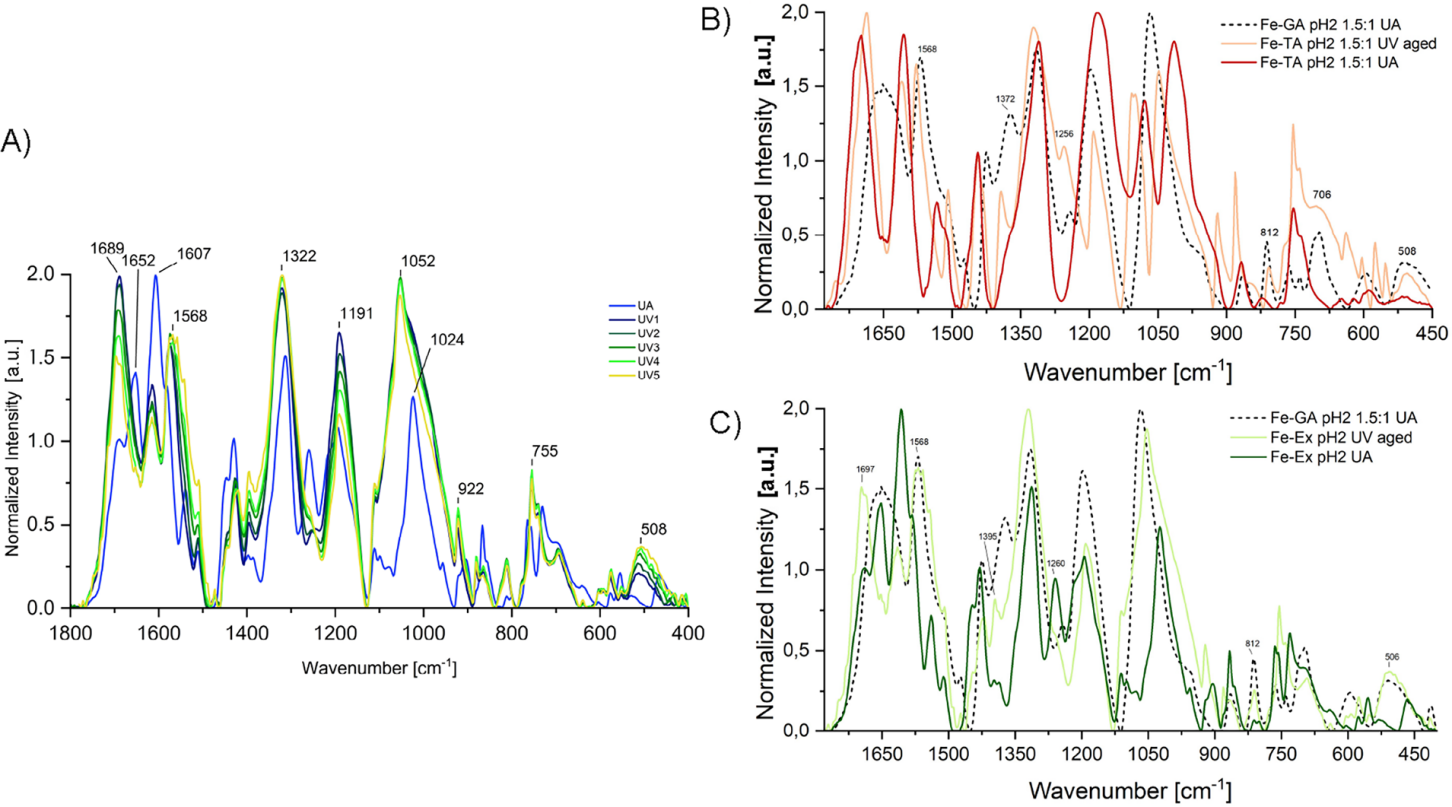
(A) FTIR spectra of the Fe-Ex pH 2 subset during UV aging. (B) Overlay of FTIR spectra of unaged (UA) and UV-aged (750 h exposure) Fe-TA pH 2 1.5:1 samples. (C) Overlay of FTIR spectra of unaged and UV-aged (750 h exposure) Fe-Ex pH 2 samples. In panels B and C, the spectrum of Fe-GA pH 2 1.5:1 (UA) is also shown as a dotted line, supporting the hypothesis of a hydrolytic process followed by structural reorganization, since gallic acid is likely formed through hydrolysis and may subsequently reassociate into iron–gallate complexes.

**Table 4:**
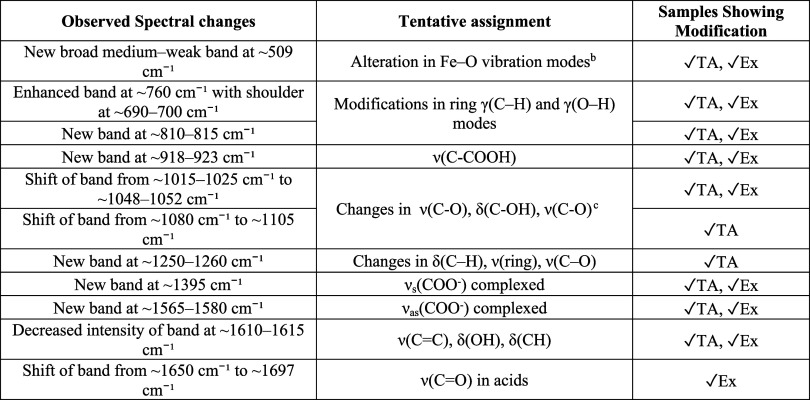
Summary of the Spectral Changes Observed during the UV Ageing of the Subsets Fe-TA pH2 1.5:1 and Fe-Ex pH2, Possibly Associated to Hydrolytic Processes^5,8,30,31,55–57^ ,^a^

a In the last column, for brevity TA and Ex denote UV-aged Fe–TA pH2 (1.5:1) and UV-aged Fe–Ex pH2. See also Supporting Information F.S12; ν: stretching vibration, δ: bending vibration (in-plane), γ: bending vibration (out of plane).

b Alternatively, this feature may correspond to an enhancement of out-of-plane ring bending modes.

c This change may also arise from modifications in δ­(C–H), ν­(ring),^30^ or in the symmetric and asymmetric ν­(C–O–C) vibrations of aryl phenolic esters.^55^

As previously noted, a single EPR parameter—the line width in the *g* ≈ 2 region—is influenced by multiple overlapping factors, complicating its interpretation. Similarly, FTIR spectra, which are characterized by a large number of peaks, poorly resolved and often overlapping, are also not straightforward to interpret. Moreover, hydrolytic processes are likely to take place, but their occurrence does not exclude the possibility of changes induced solely by variations in water content during aging. Therefore, the proposed interpretations should be further investigated through more targeted and in-depth studies.

In the Fe-Ex samples, the Raman data are consistent with the trends previously described for both the Fe-GA and Fe-TA series. The derived B/A and D/A parameters increase upon UV exposure, reflecting the progressive oxidation of phenolic moieties. The extent of these changes varies with the pH, as will be discussed later. In parallel, the distribution of A-band positions gradually shifts to lower wavenumbers, likely indicating an increase in the amorphous character of the particle microstructure. Both effects are relevant during UV aging, whereas RH aging does not induce comparably significant changes in these parameters. Interestingly, the FTIR results suggest that hydrolytic processes may also be initiated at early stages of UV exposure in this system (Figure 11). In fact, as observed for FeTA at pH 2, FeEx at pH 2 also shows, upon UV aging, pronounced spectral modifications (most notably in the 850–400 cm^–1^ range, with blue shifts of the bands near 1024 and 1540 cm^–1^ and an overall profile change between 1750 and 1500 cm^–1^) which further support this hypothesis (Figure 11C, see also Supporting Information Figures F.S12 and F.S13). However, unlike what was observed for Fe-TA pH2, the pronounced changes in the IR spectral profile are not accompanied by significant shifts in the Raman A band or by a decrease in the line width of the main CW-EPR signal at *g* ≈ 2. It is important to note that previous studies have shown that OG extracts are primarily composed of gallotannins (GT) with a lower degree of polymerization (a lower degree of galloyl esterification) compared to TA.^53^ Moreover, as natural botanical extracts, they likely contain a more chemically complex mixture, including free sugars and other low-molecular-weight polyphenols.^54^ Within this context, it can be hypothesized that hydrolytic processes may also occur in Fe-Ex, although to a minor extent. In any case, the predominant effect appears to be a progressive weakening of the metal–polyphenol interactions (see Figure F.S13 in the Supporting Information).

As expected, a very similar aging behavior was observed for the model inks, but in this case, possibly due to the signals related to AG, the FTIR results do not show clear evidence of hydrolytic processes.

As outlined in the introduction, one of the main degradation pathways of IGI contributing to the overall deterioration of manuscripts is the oxidation of phenolic moieties. This process, as well documented in the literature,^1,7,58,59^ occurs via a Fenton-like reaction, leading to the reduction of Fe^3+^ to Fe^2+^. This redox conversion is known to cause ink fading, color shifts, and the formation of brownish haloes. Moreover, the reaction mechanism involves the formation of reactive radical species, which are known to play a significant role in the degradation of ink support.^3,11,21^ In the present study, the Raman-derived B/A parameter is used as an indicator of this oxidative process, allowing for a comparative evaluation across the different sample sets.

To gain insight into the extent of this process, one can consider the percentage increase in the mean values of the B/A parameter following UV exposure, which—as previously noted—was the aging protocol that had the most pronounced effect on this parameter. Although this approach provides only a rough estimation, it nonetheless highlights the critical role of ligand structure in the oxidation process. As will be further discussed, the extent of the B/A increase is strongly influenced by pH. However, even at a fixed pH of 2, a clear difference emerges between the Fe-GA and Fe-TA subsets: for Fe-GA, the increase ranges from 11.04 to 14.47%, whereas for Fe-TA, it spans from 16.93 to 94.99%. The Fe-Ex subset prepared at pH 2 exhibited a percentage increase in this parameter of 21.57%, which may be considered intermediate relative to the Fe-GA and Fe-TA subsets (Table 5, see also Table T.S1 in Supporting Information).

**Table 5:** Percentage Increase of the Raman-Derived B/A Parameter in the Different Subsets of Fe-GA, Fe-TA, and Fe-Ex Prepared at pH 2^a^

ligand	subset	increase of B/A (%)
GA	Fe-GA pH 2 – 0.8:1	11
	Fe-GA pH 2 – 1:1	14
	Fe-GA pH 2 – 1.5:1	13
	Fe-GA pH 2 – 5:1	12
TA	Fe-TA pH 2 – 1:1	62
	Fe-TA pH 2 – 1.5:1	17
	Fe-TA pH 2 – 3:1	30
	Fe-TA pH 2 – 5:1	95
Ex	pH2	22

a The increase was calculated as the relative difference between the minimum and maximum mean values of the B/A parameter within each subset. While this is not a statistically robust metric, it provides a useful basis for comparison across datasets. For further details, refer to Table T.S1 in the Supporting Information.

This evidence supports the idea of a strong influence of the ligand structure not only in terms of type of possible degradation patterns, as commented before, but also in terms of the extent of the common oxidative process affecting the ligand phenolic moieties in IGI.

Finally, it is worth noting that the percentage increase in the mean B/A values for the model ink set appears lower than that observed for the Fe-Ex samples, ranging from 6.72 to 12.66%, compared to 13.01–21.57% for the Fe-Ex samples (see also Table T.S1 in the Supporting Information). While a direct and accurate comparison is hindered by the substantial differences in the preparation protocols of these two sample sets, the results nonetheless suggest that the presence of AG in the IGI formulation may contribute to reducing or at least slowing down the oxidation processes.

A final practical consideration concerns the observation of organic radicals in iron-polyphenolic systems. As previously noted, the detection of radical species is hindered by the relatively high abundance of paramagnetic Fe^3+^ cations per unit mass or volume of sample. Consequently, organic radicals (*g* ≈ 2.0027–2.0028) have only been detected in systems employing TA as a ligand (see Figure 5). In these cases, the high molecular weight of the ligand results in a reduced concentration of iron per unit mass, thereby facilitating the detection of radical signals. Nonetheless, further investigations are required to elucidate the mechanisms of radical formation and evolution in such systems. The development of improved methodologies tailored to this purpose is also warranted.

### 3.4 Role of the pH

Previous studies have already emphasized the role of pH in determining the structure of iron-polyphenolic complexes, both in terms of stoichiometry and coordination geometry.^7,8,31^ The results obtained here highlight the importance of pH in influencing both the nature and the extent of degradation processes. Consistently, Raman data from Fe-GA, Fe-TA, and Fe-Ex sets of samples indicate that lower pH conditions generally promote more pronounced degradation. For instance, the percentage increase in mean B/A values, the Raman-derived parameter used to assess phenolic moiety oxidation, progressively decreases with increasing pH (see also Table T.S1 in the Supporting Information). Samples prepared with ligand-containing solutions buffered at pH 2 exhibited the greatest increase in this parameter, whereas those prepared at pH 6 demonstrated enhanced stability under both UV and RH aging. This observation aligns with previous findings showing that iron-polyphenolic complexes formed at pH 2 are characterized by weaker metal–polyphenol interactions, as evidenced by CW-EPR measurements (Figure F.S3 in the Supporting Information). Additionally, the pH dependence of the Fenton-like reaction represents another key factor consistent with these results.^25,60,61^

Similarly, the iron–polyphenolic complexes formed under stronger acidic conditions showed a higher tendency for the A-band distribution to shift toward slightly lower wavenumbers. In particular, the shift was observed for Fe-GA samples at pH 2 and 4 and for Fe-Ex at pH 2, indicating a greater tendency toward amorphization of the solid microstructure compared to samples prepared at higher pH (Figure 12).

**Figure 12:**
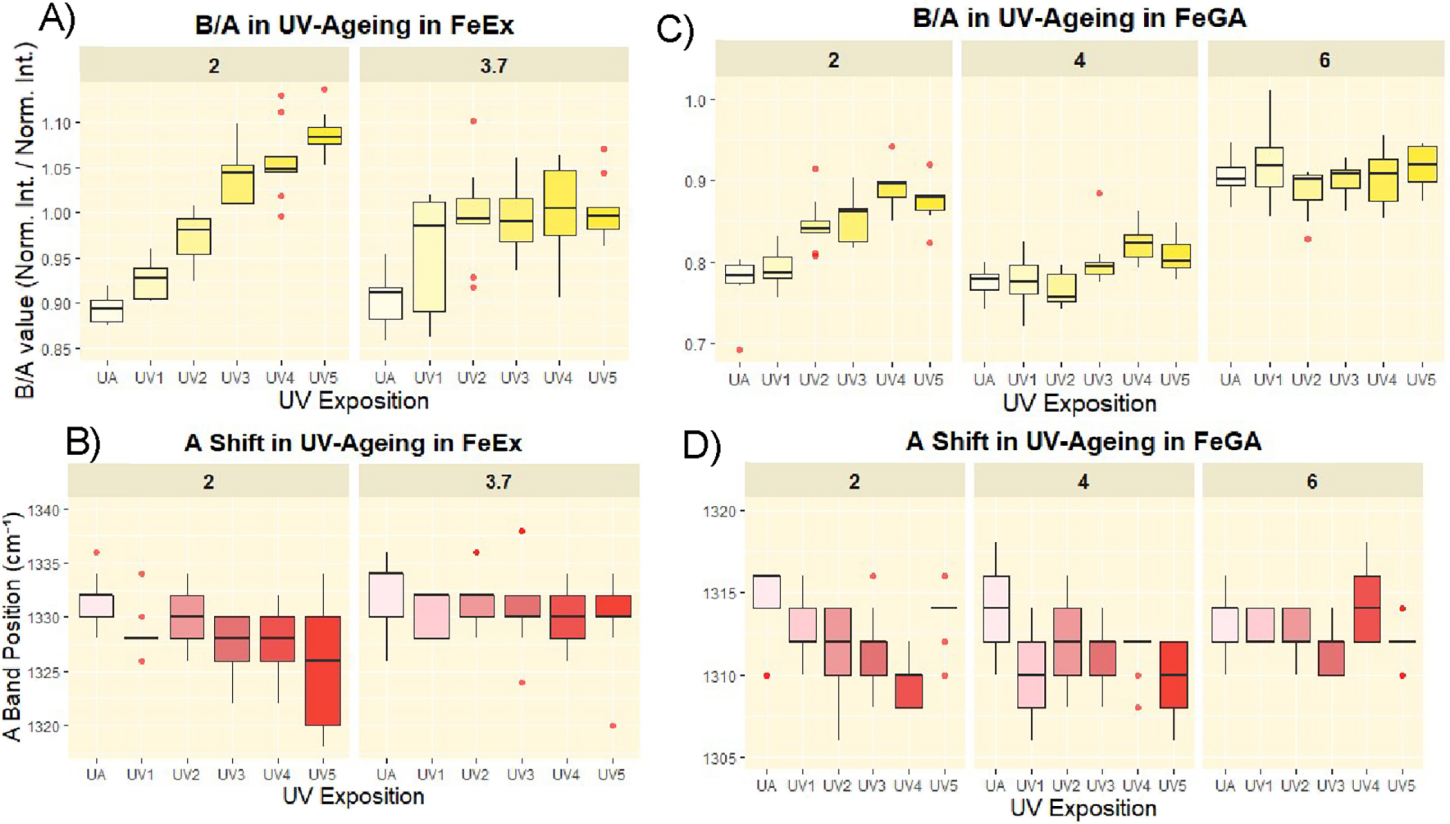
Boxplots illustrating the influence of pH on the increase of the Raman-derived B/A parameter and the shift of the A band during UV aging. Panels (A,B) correspond to the Fe-Ex sample set, while panels (C,D) refer to the Fe-GA subsets prepared at pseudo-stoichiometric Fe-to-GA ratios (1:1 for pH 2 and pH 4, and 1:2 for pH 6).

In the previous paragraph, the occurrence of hydrolytic processes was mentioned as a relevant degradation process observed in the Fe-TA and Fe-Ex sets under UV exposure. It should be highlighted here that these processes were indeed observed just in the subset prepared at pH 2 (Figure 12, see also Figure 11). The mechanisms underlying ligand hydrolysis, and the consequent potential complexation of iron cations with newly freed phenolic—and possibly carboxylic—moieties, should be further explored in future studies. However, the findings reported here suggest that this degradative process is catalyzed under strongly acidic conditions.^50,62^

### 3.5 Role of the Fe Concentration

Understanding the role of the iron-to-ligand ratio in the degradation of iron–polyphenolic complexes remains challenging due to practical limitations in sample preparation, particularly in controlling stoichiometry and isolating solid precipitates. At room temperature, Fe^2+^ complexes form rapidly, which, while advantageous for synthesis, often leads to amorphous and potentially heterogeneous products. This complicates the investigation of the parameters influencing both complex formation and aging. Notably, as previously demonstrated,^8^ the iron-to-ligand ratio appears to play a limited role in defining the structure of these complexes. This consideration is crucial when evaluating its influence on degradation processes in Fe-GA and Fe-TA (each synthesized at four different iron-to-ligand molar ratios), as well as in model inks (prepared with three Fe:OG:AG ratios). In all cases, iron’s role in degradation appears heterogeneous, indicating a strong interplay between iron-to-ligand ratio, pH and polyphenolic structure in the degradative forces.

In Fe-GA for instance, the acquired data are not statistically robust enough to suggest any specific pattern: the increasing of the B/A and D/A derived Raman parameters and the A band shift, considering the variability of these data, do not support any specific trend in most of the cases, in both types of aging protocols (see also Figure F.S7 in the Supporting Information). Similarly, EPR and FTIR do not highlight a statistically significant difference among the patterns observed for different iron-to-ligand ratios.

In Fe-TA, the initial differences and subsequent percentage increases in Raman B/A and D/A parameters suggest that, in most cases, higher iron content is associated with a greater or faster oxidation of phenolic moieties. However, this trend is not fully supported by EPR and FTIR data, which indicate a weaker correlation between iron-to-ligand ratio and the extent of oxidation and degradation. While Raman results point to a possible positive correlation, the overall findings suggest a more heterogeneous degradation behavior. Notably, in both Fe-GA and Fe-TA, shifts in the Raman A band—when significant—are more pronounced at low to intermediate iron-to-ligand ratios. For example, in Fe-TA at pH 2, signs of a hydrolytic process (as previously discussed) were observed at 1.5:1 and 3:1 ratios, less evident at 1:1, and absent at 5:1, based on Raman, FTIR, and EPR data. In contrast, the model ink aging results present a more complex scenario: although B/A and D/A ratios show comparable increases, the A-band Raman shift is more pronounced at higher iron concentrations (Figure 13).

**Figure 13:**
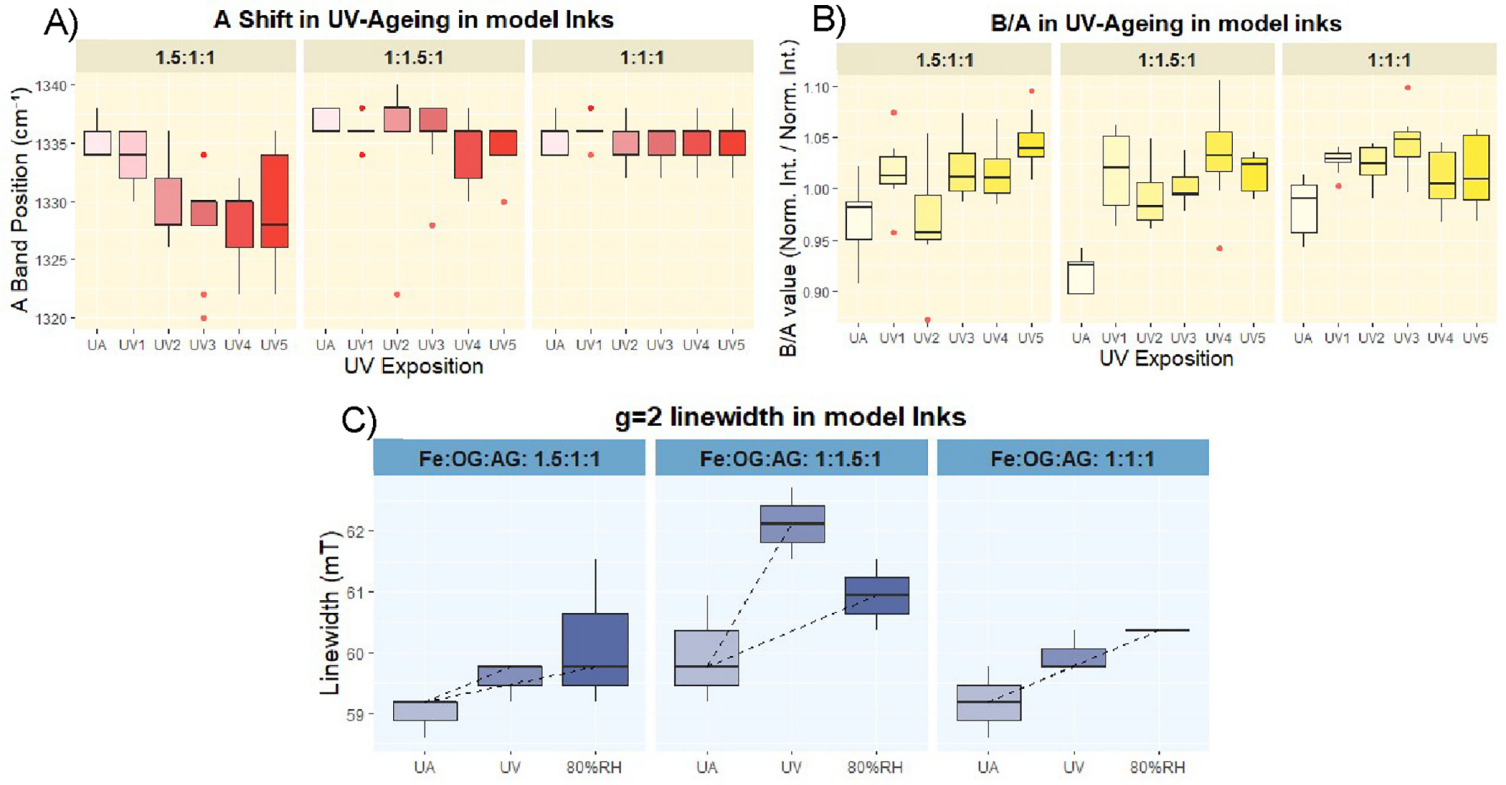
(A, B) Boxplots showing the shift of the Raman A-band and the increase of the B/A parameter during UV aging of the three model inks investigated. (C) Boxplots illustrating the increase in the line width of the EPR signal at *g* ≈ 2 as a result of aging of model inks. Data from both UV and relative humidity (RH) aging conditions are presented.

## 4 Conclusions

This study investigated the influence of polyphenolic ligand structure, pH, and iron-to-ligand ratio on the aging of iron–polyphenolic complexes. The multi-analytical approach provided evidence that the ligand structure and pH are the primary factors influencing degradation patterns. Specifically, oxidation of phenolic moieties (mainly driven by Fenton-like reactions involving Fe^3+^ reduction) was found to be more pronounced under strongly acidic conditions. In such environments, hydrolytic processes were also hypothesized, particularly in complexes prepared with TA and OG extracts. It was highlighted that, in the degradation of iron–polyphenolic complexes alone (i.e., without the support), UV exposure had an overall stronger impact than RH, which is instead known to be a major degradative agent for ink supports. The formation of SPIONs of varying sizes was observed and may contribute to additional degradation pathways.

The methodology allowed for efficient comparison across data sets and also highlighted critical aspects requiring further investigation. For instance, FTIR and Raman data suggest the occurrence of hydrolytic processes, which would benefit from Mass Spectrometry based analyses to better elucidate their mechanisms and the roles of pH and iron concentration. Similarly, the formation of organic and potentially also other types of radicals warrants further study to identify their nature and behavior. The influence of iron concentration on the degradation of iron–polyphenolic complexes remains unclear and would require more controlled synthetic procedures to be properly assessed. Despite these open questions, the findings emphasize the importance of preparing well-designed and adequate representative IGI models for studying their degradation. Such models are essential not only to evaluate common degradation phenomena (such as phenolic oxidation) but also to capture the full range of possible degradation pathways occurring in IGI. A thorough understanding of the full range of these degradative phenomena, on one side contributes to the study and preservation of IGI themselves, preventing issues such as discoloration and other alterations in color. On the other side, and often more importantly, it provides a solid basis for future investigations on the degradation of manuscripts as a whole, as to say the influence of IGI degradation on accelerating the overall deterioration of their supports, such as parchment and paper.

## Supplementary Material



## Notes

Additional data such as Raman, FT-IR, and EPR spectra and their derived elaborations (PDF)
