# Supplementary material for: How the Variability of Iron-Polyphenolic Complexes Affects the Degradation of Iron-Gall Inks: A Multi-Analytical Study
Source: ACS Omega. 2026 Jan 21;11(4):5639–54. doi: 10.1021/acsomega.5c09386 (PMC12878493; doi:10.1021/acsomega.5c09386)
Supplement: Supplementary file 1 [file ao5c09386_si_001.pdf]

Supporting Information

How the variability of iron-polyphenolic complexes affects the degradation of iron-gall inks: a multi-analytical study

Salvatore Caterino[1*], Iulia Maria Caniola[1], Marc Pignitter[2], Alfonso Zoleo[3], Santiago Sanchez-Cortes[4], Katja Sterflinger[1], Federica Cappa[1]

[1] Institute of Natural Sciences and Technology in the Arts, Academy of Fine Arts of Vienna, Vienna, Austria.
[2] Institute of Physiological Chemistry, Faculty of Chemistry – University of Vienna, Vienna, Austria.
[3] Department of Chemical Sciences – University of Padua, Padua, Italy.
[4] Instituto de Estructura de la Materia (CSIC), Madrid, Spain

* corresponding author
s.caterino@akbild.ac.at

F.S1 Variability of the Raman-derived parameter F_{max}/A due to UV ageing in FeTA pH4

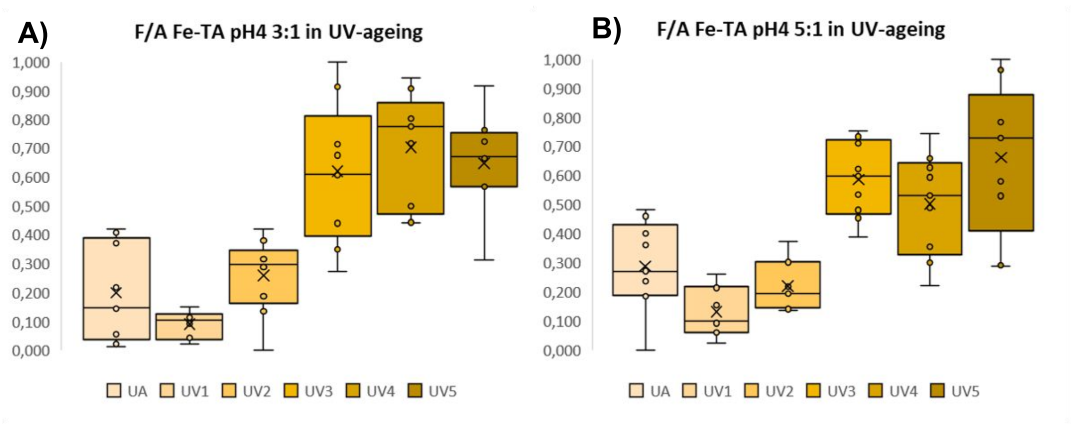

Boxplots showing the variation of the Raman spectral parameter F/A (defined as the peak intensities ratio between the maximum of the spectral region 400–650 cm⁻¹ and the height of the A band) as a result of UV ageing in (A) FeTA pH4 3:1 and (B) FeTA pH4 5:1 subsets. Before plotting, the values were secondarily normalized between 0 and 1 to emphasize the observed changes.

F.S2 FTIR spectral profile changes due to UV-ageing in Fe-Ex and model inks

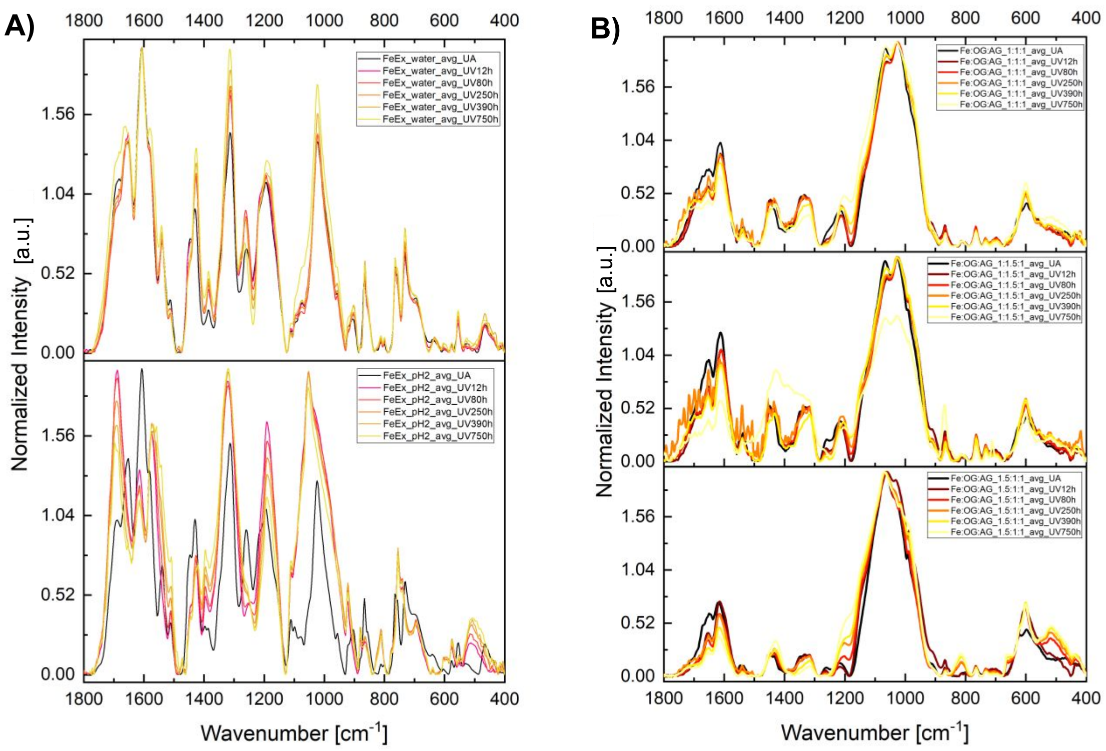

FTIR spectra related to the UV-ageing of A) Fe-Ex and B) model inks. The Fe-Ex pH 2 set (bottom panel in A) will be discussed separately in the text. Overall, the spectra display only minimal changes in their profiles.

F.S3 Variability of the linewidth of the g=2 and g=4.3 signals within the FeGA and FeTA datasets

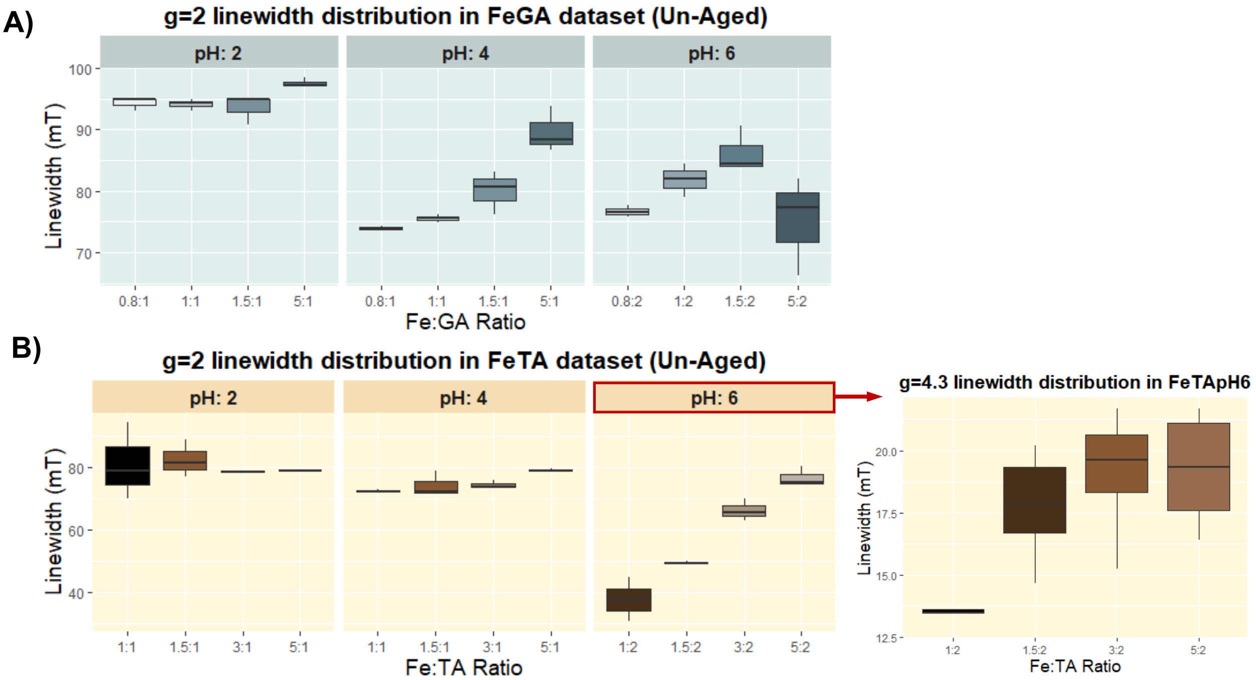

A) Boxplots showing the variability of the linewidth of the signal at g≈2 across the Fe-GA dataset. B) Boxplot showing the distribution of linewidth of the signal at g≈2 across the Fe-TA dataset and, on the right, the distribution of linewidth of the signal at g≈4.3 across the Fe-TA pH6 subset

F.S4 Linewidth opening due to ageing in FeGA dataset

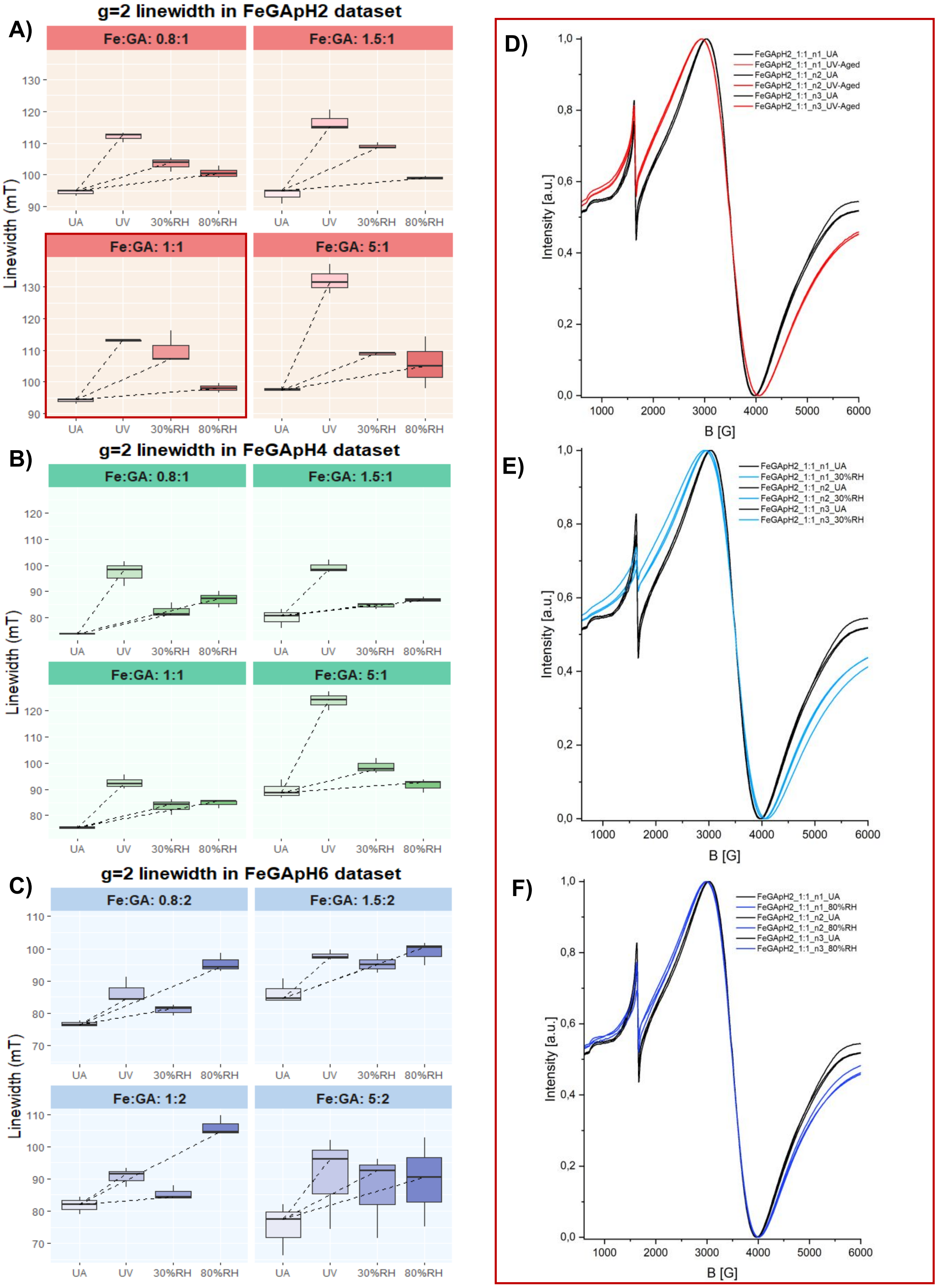

A–C) Boxplots showing how the linewidth of the g ≈ 2 signal increases after artificial ageing in the Fe-GA dataset. D–F) CW-EPR spectra from the FeGA pH2 1:1 subset. The spectra are reported as an emblematic example to highlight the opening of the g ≈ 2 signal after ageing under D) UV light, E) 30% relative humidity, and F) 80% relative humidity.

F.S5 Signal associated with the peculiar SPION system in the FeEx pH2 set aged at 30% RH

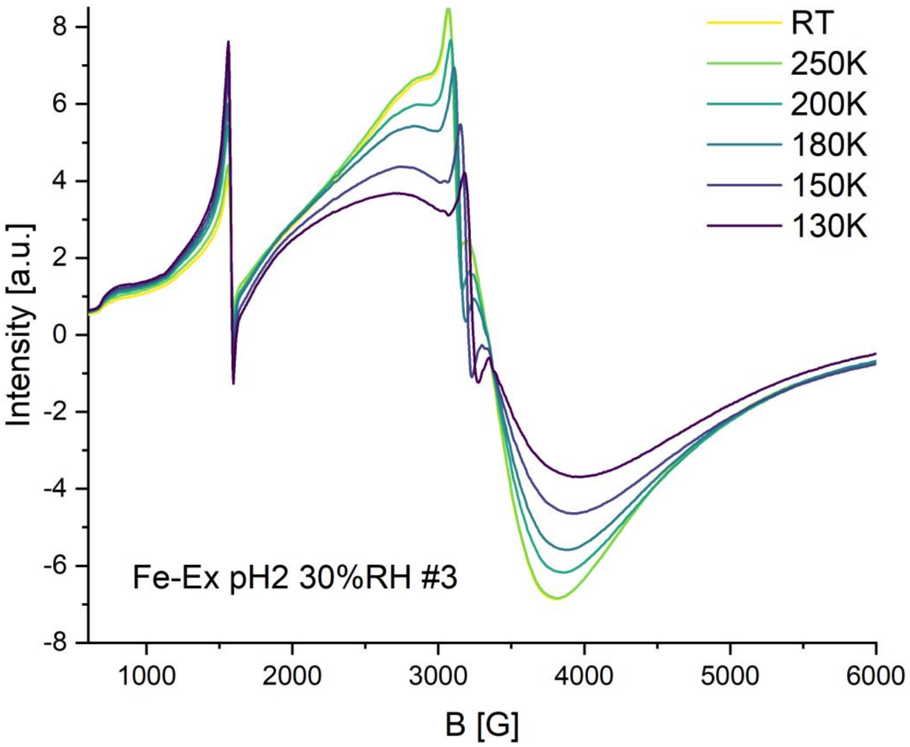

The CW-EPR spectra acquired at variable temperatures between RT and 130 K shows that the sharp signal around 3000 G is actually related to a SPION: the field shift on changing the temperature is a clear indication for it. For most of SPION, the opposite trend is expected, i.e., the signal shifts generally to a lower field on lowering the temperature, and line broadening is observed. However, the observed trend has been also observed, and normally attributed to systems dominated by magnetic anisotropy contributions. Actually, the interplay between magnetic anisotropy, applied field, and temperature, can lead to complicated EPR spectra of SPION systems.

F.S6 Variability of the Raman-derived parameter B/A and D/A due to RH ageing in both FeGA and FeTA sets

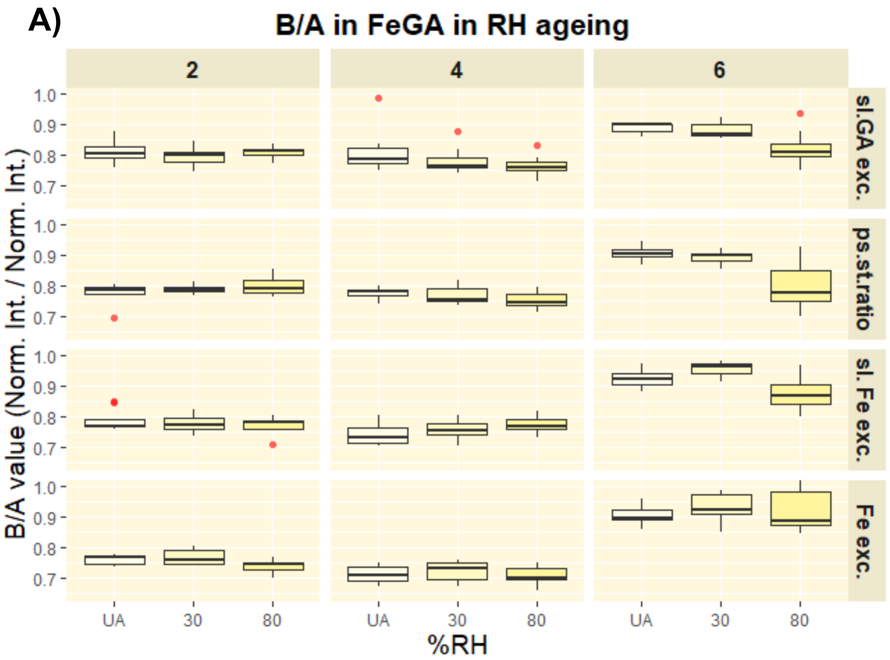

B)

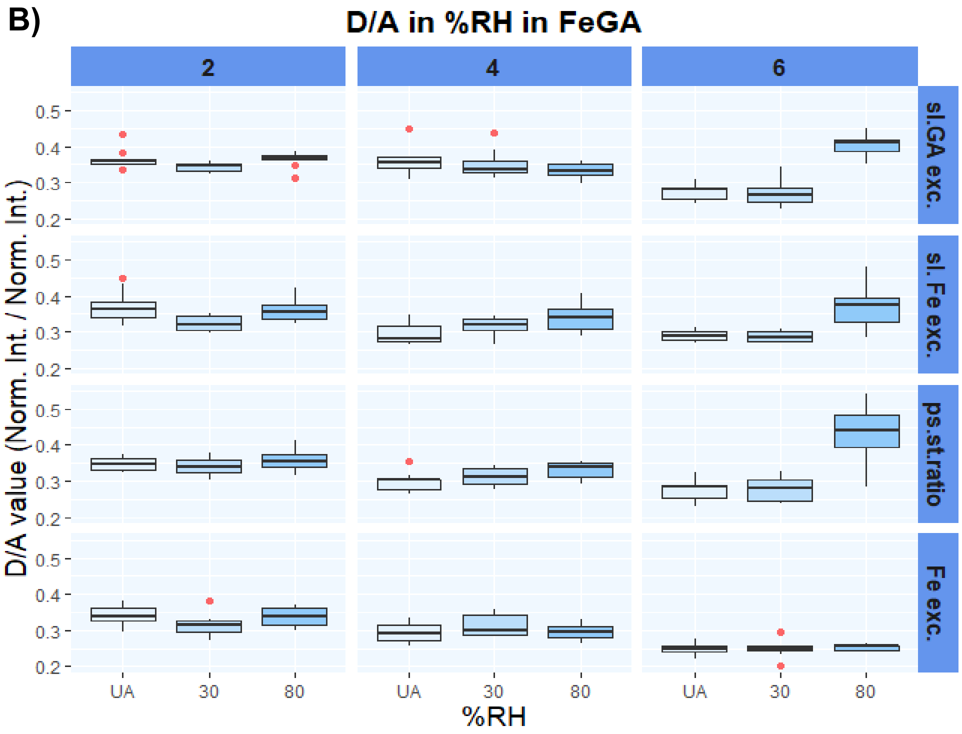
D/A in %RH in FeGA
2
4
6
sl.GA exc.
sl. Fe exc.
ps.st.ratio
Fe exc.
0.5
0.4
0.3
0.2
D/A value (Norm. Int. / Norm. Int.)
UA
30
80
%RH

C)

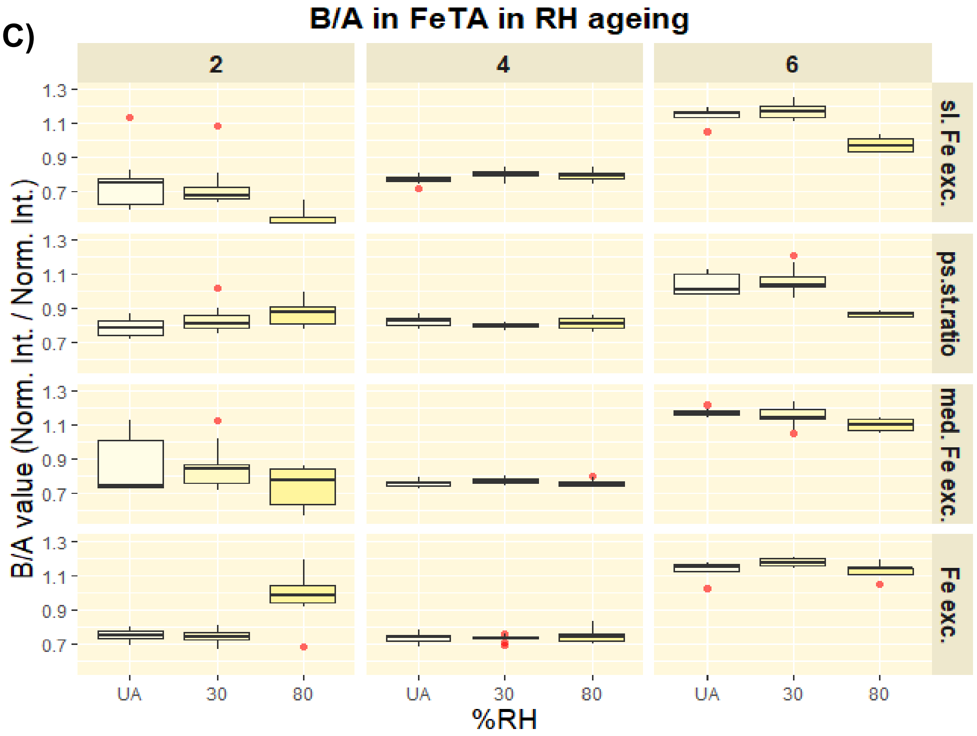
B/A in FeTA in RH ageing
2
4
6
sl. Fe exc.
ps.st.ratio
med. Fe exc.
Fe exc.
1.3
1.1
0.9
0.7
B/A value (Norm. Int. / Norm. Int.)
UA
30
80
%RH

D)

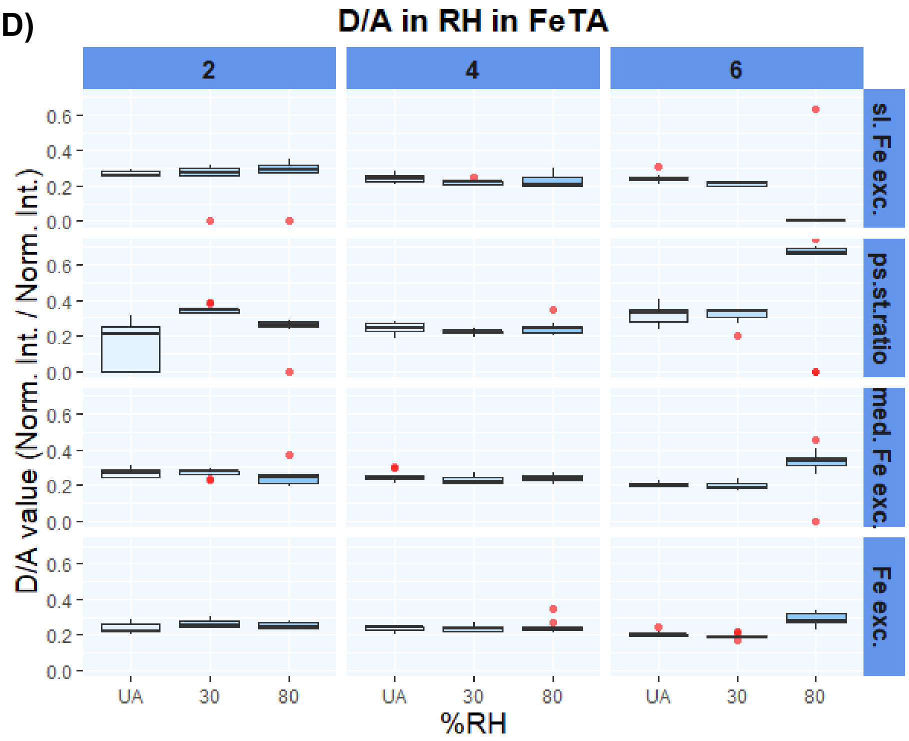

Boxplots showing the variation of Raman spectral parameters due to RH ageing in the FeGA and FeTA datasets: (A) B/A variation due to RH ageing in FeGA set, (B) D/A variation due to RH ageing in FeGA set, (C) B/A variation due to RH ageing in FeTA set, (D) D/A variation due to RH ageing in FeTA set.

Outliers (automatically identified as values beyond 1.5×IQR from Q1 or Q3) are marked in red. For clarity, Fe:GA ratios are grouped as follows (panels A and B): sl.GA exc. (0.8:1 for pH2/pH4; 0.8:2 for pH6), ps.st.ratio (1:1, 1:2), sl. Fe exc. (1.5:1, 1.5:2), and Fe exc. (5:1, 5:2). Fe: TA ratios are grouped as follows (panels C and D): ps.st.ratio (1:1 for FeTA-pH2 and pH4, 1:2 for FeTA-pH6), sl. Fe exc. (1.5:1 for FeTA-pH2 and pH4, 1.5:2 for FeTA-pH6), med. Fe exc. (3:1 for FeTA-pH2 and pH4, 3:2 for FeTA-pH6) and Fe exc. (5:1 for FeTA-pH2 and pH4, 5:2 for FeTA-pH6).

F.S7 Variability of Raman spectra on Fe-GA samples under UV Artificial Ageing

A)

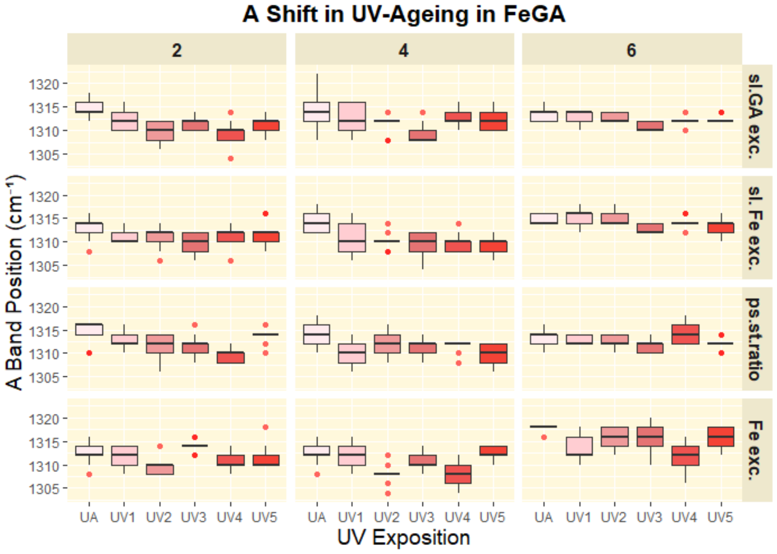

B)

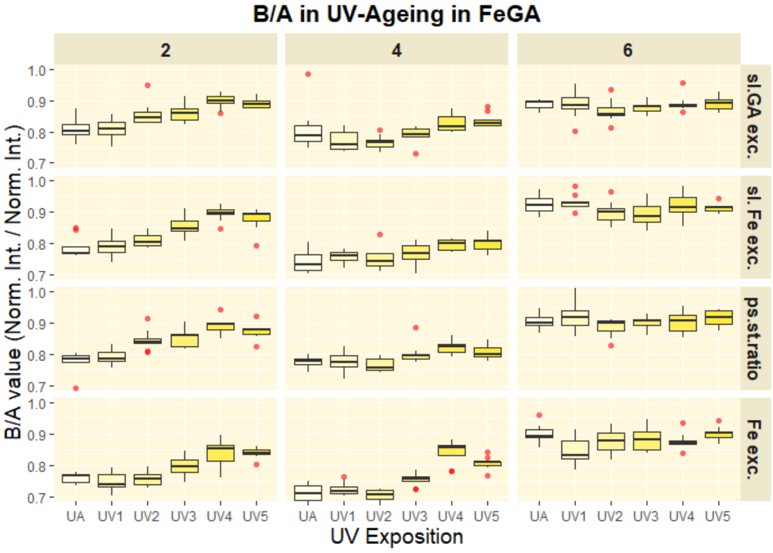

C)

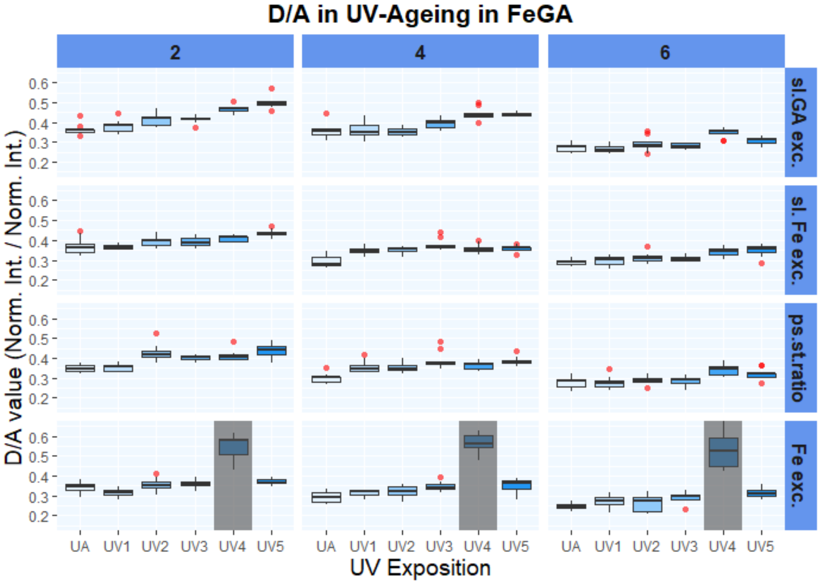

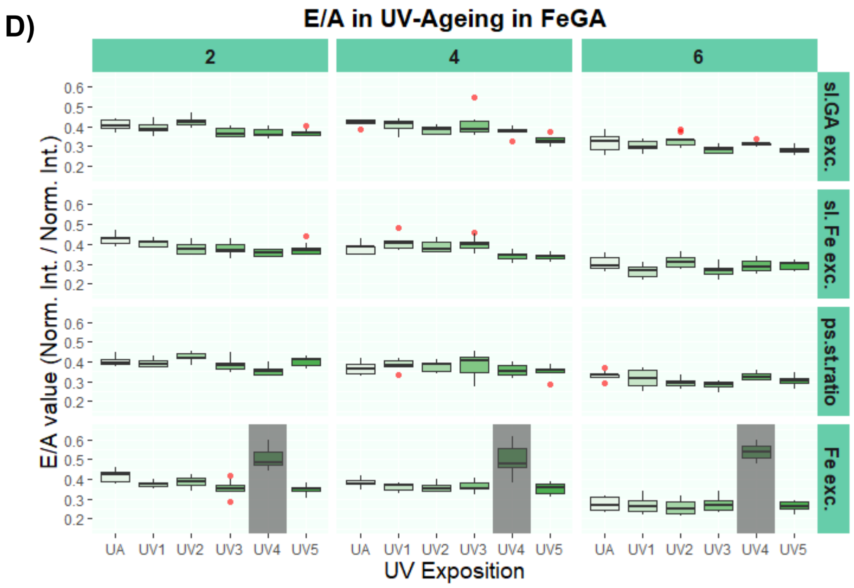

Boxplots showing the variation of Raman spectral parameters due to UV ageing: (A) A band position, (B) B/A, (C) D/A and D) E/A. Outliers (automatically identified as values beyond 1.5×IQR from Q1 or Q3) are marked in red.

For clarity, Fe:GA ratios are grouped as follows: sl.GA exc. (0.8:1 for pH2/pH4; 0.8:2 for pH6), ps.st.ratio (1:1, 1:2), sl. Fe exc. (1.5:1, 1.5:2), and Fe exc. (5:1, 5:2). In panels C and D, the boxplots for Fe exc. UV4 (390 h of exposure) are obscured, as the corresponding values deviate from the overall trend due to poor spectral quality—likely caused by an instrumental issue during acquisition. This artefact had limited impact on “A position” and “B/A” parameters, which are derived from intense bands and thus less sensitive to noise.

F.S8 Variability of the Raman A band position due to artificial ageing in FeTA

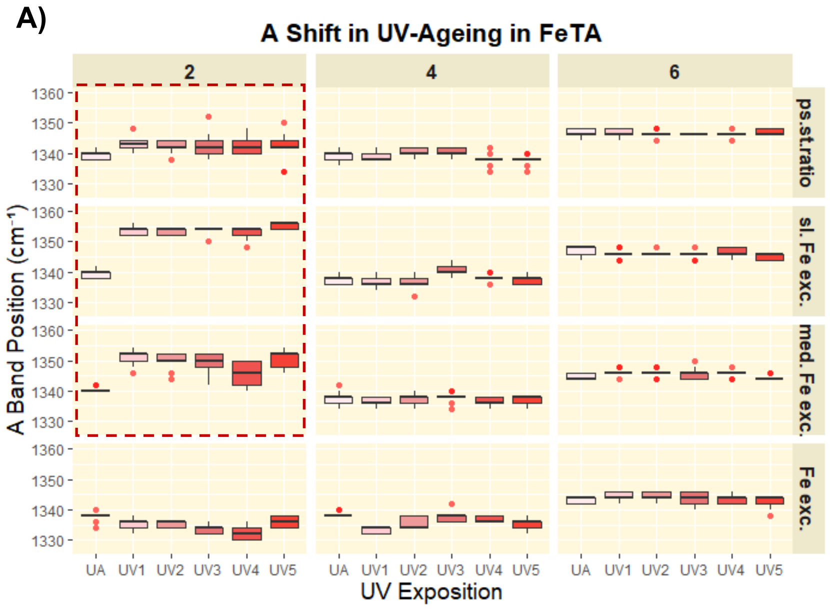

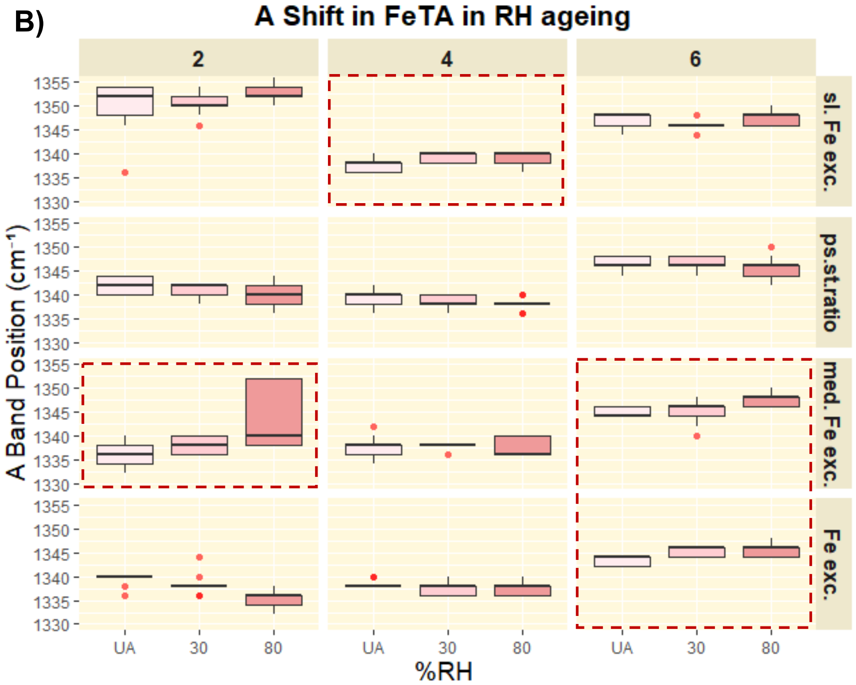

Boxplots showing the variation of Raman A band position in FeTA due to: (A) UV exposure, (B) RH ageing. Outliers (automatically identified as values beyond 1.5×IQR from Q1 or Q3) are marked in red. The dotted-line square in panel A highlights the most intense A band shifts observed during the UV ageing across the FeTA whole dataset. Similarly, in panel B, dotted-line squares highlight the pH and Fe:TA conditions in which a shift towards higher wavenumbers can be observed, even if with a less strong extent.

For clarity, Fe:TA ratios are grouped as follows: ps.st.ratio (1:1 for FeTA-pH2 and pH4, 1:2 for FeTA-pH6), sl. Fe exc. (1.5:1 for FeTA-pH2 and pH4, 1.5:2 for FeTA-pH6), med. Fe exc. (3:1 for FeTA-pH2 and pH4, 3:2 for FeTA-pH6) and Fe exc. (5:1 for FeTA-pH2 and pH4, 5:2 for FeTA-pH6).

F.S9 Variability of the EPR g≈2 signal linewidth due to artificial ageing in FeTA

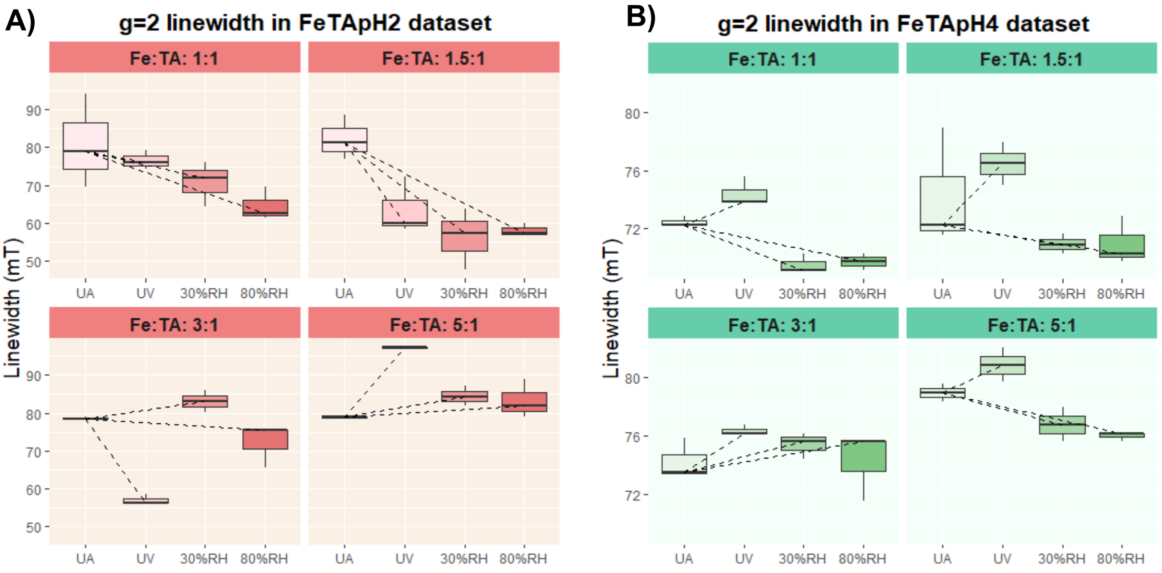

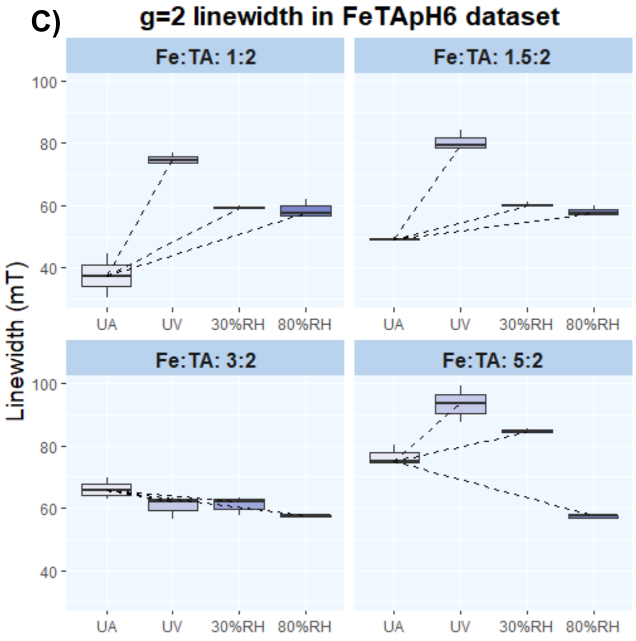

Boxplot showing the variability of the g≈2 EPR signal linewidth due to artificial ageing in (A) FeTA pH 2, (B) FeTA pH 4 and (C) FeTA pH 6. The comparison highlights how in certain conditions and in particular in FeTA pH 2, the signal linewidth decreases. As mentioned in the paper, this experimental evidence can be associated to the loss of water during the ageing process or to a partial hydrolysis of the ligands.

F. S10 Acid-catalysed and UV-triggered hydrolysis of large polyphenolic ligands

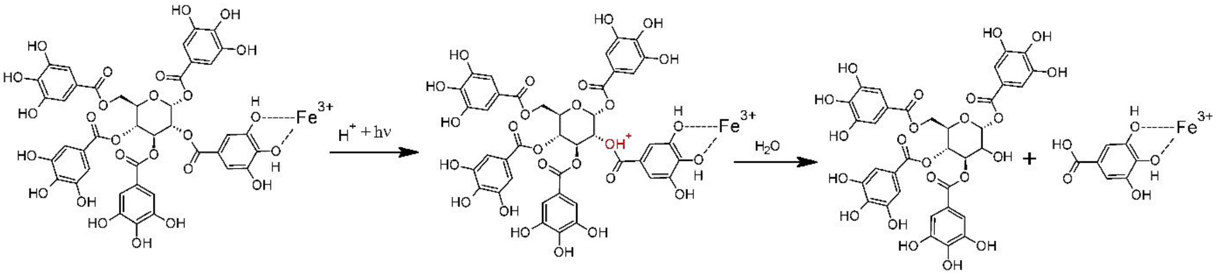

Example of a possible acid-catalysed hydrolysis mechanism for a Fe-Penta galloyl glucose (PGG) complex. The scheme illustrates how the cleavage of the ester bond (sugar–galloyl moiety) can be triggered by acidic conditions and UV light, leading to the formation of Fe–GA acid complexes and a potential rearrangement of the overall complex structure.

F.S11 Variability of the FTIR spectral profiles due to UV ageing in FeTA

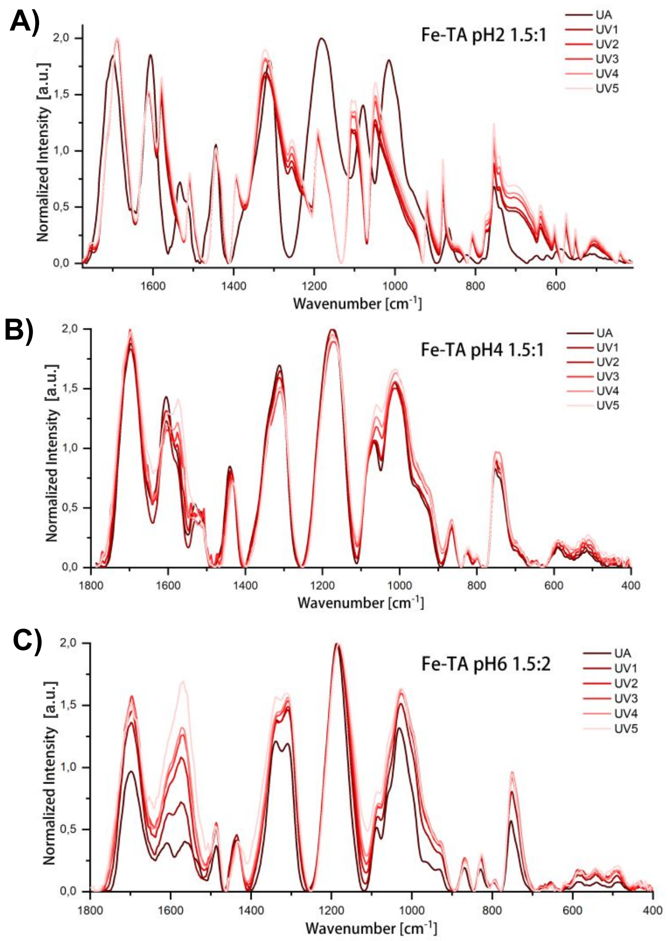

ATR-FTIR spectra highlighting UV-induced modifications in the FeTA sets: (A) pH 2 1.5:1, (B) pH 4 1.5:1, and (C) pH 6 1.5:2. The pH 2 samples undergo the most marked spectral alterations, not only in band intensity but also in the appearance of new signals and distinct shifts of major bands (notably within 1600–1000 cm^{-1}). Comparable behavior is found in other subsets, particularly when contrasting FeTA prepared at pH 2 1:1 with those at pH 4 1:1 and pH 6 1:2.

F.S12 FTIR spectral changes possibly associated with hydrolysis of the polyphenolic ligands

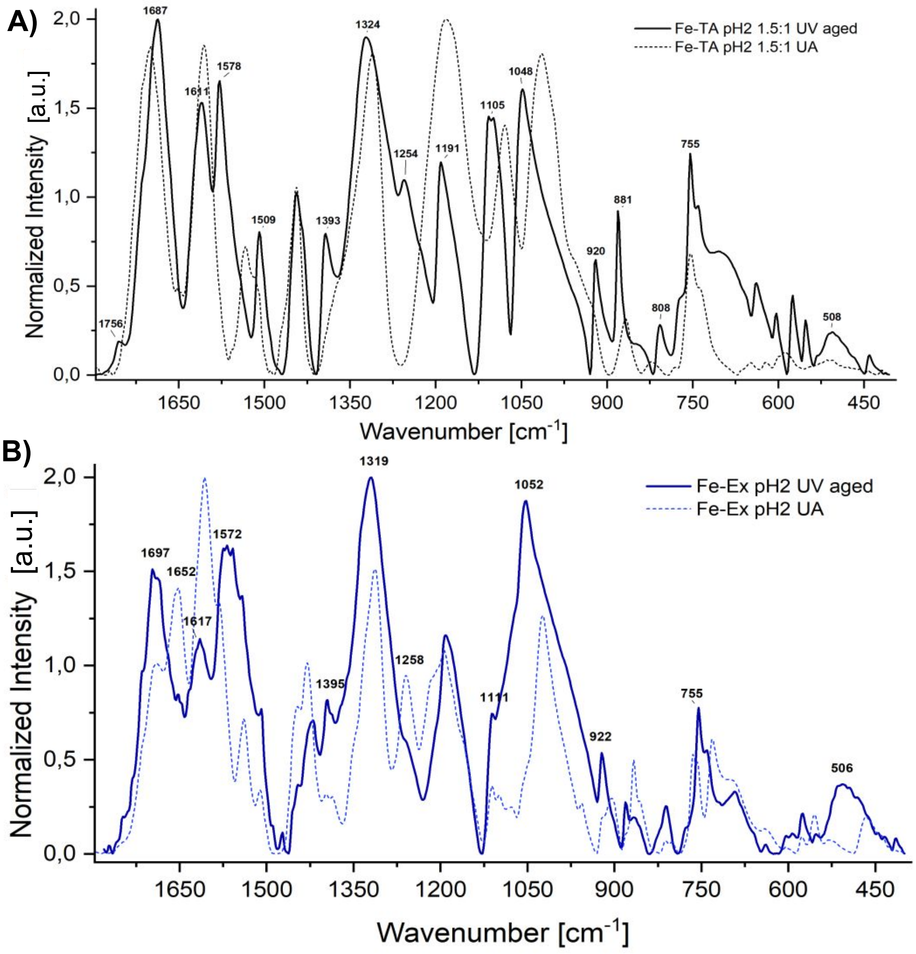

FT-IR spectra of A) UA and UV-Aged (750h) FeTA pH2 1.5:1 and B) UA and UV-Aged (750h) FeEx pH2. In the spectra the major changes possibly associated with an hydrolyzation pattern (commented in Tab.4 of the main paper) are visible.

F.S13 Variability of the EPR g≈2 signal linewidth due to artificial ageing in FeEx

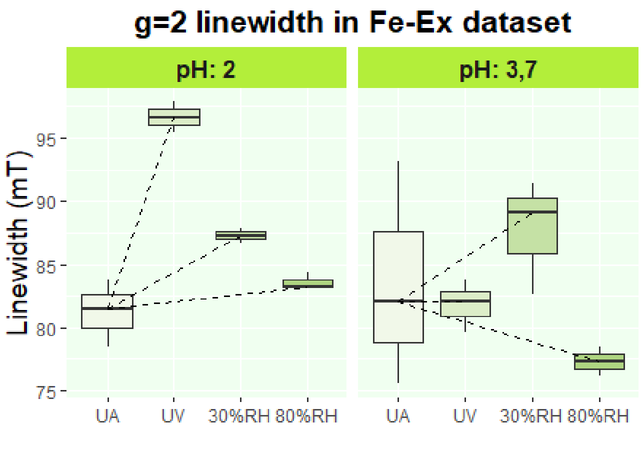

Boxplot showing the broadening of the g≈2 EPR signal linewidth induced by artificial ageing in the FeEx samples, in agreement with the general weakening of coordination bonds discussed in the paper.

Table T.S1

Summary of the B/A Raman parameter variations induced by UV ageing. For each subset, the minimum and maximum mean values are reported along with their corresponding standard deviations (formatted as mean ± SD). The percentage increase between these values was also calculated, with associated uncertainty estimated using the error propagation formula.

Ligand	*pH*	*iron-to-ligand*	*Min B/A mean value*	*Max B/A mean value*	*% increase*
GA	2	0.8:1	0.81 ± 0.04	0.90 ± 0.02	11 ± 6
		1:1	0.78 ± 0.03	0.89 ± 0.03	14 ± 6
		1.5:1	0.79 ± 0.04	0.89 ± 0.02	13 ± 6
		5:1	0.75 ± 0.03	0.84 ± 0.04	12 ± 7
	4	0.8:1	0.77 ± 0.02	0.84 ± 0.02	9 ± 4
		1:1	0.77 ± 0.02	0.82 ± 0.02	7 ± 4
		1.5:1	0.74 ± 0.03	0.80 ± 0.03	9 ± 6
		5:1	0.71 ± 0.02	0.84 ± 0.04	19 ± 6
	6	0.8:2	0.87 ± 0.04	0.89 ± 0.03	3 ± 5
		1:2	0.89 ± 0.03	0.92 ± 0.05	4 ± 6
		1.5:2	0.89 ± 0.04	0.93 ± 0.03	4 ± 5
		5:2	0.85 ± 0.04	0.90 ± 0.02	6 ± 6
TA	2	1:1	0.64 ± 0.02	1.0 ± 0.1	62 ± 23
		1.5:1	0.57 ± 0.06	0.67 ± 0.08	17 ± 18
		3:1	0.66 ± 0.02	0.86 ± 0.05	30 ± 8
		5:1	0.67 ± 0.03	1.3 ± 0.2	95 ± 26
	4	1:1	0.82 ± 0.03	0.99 ± 0.02	21 ± 5
		1.5:1	0.77 ± 0.03	0.97 ± 0.02	26 ± 5
		3:1	0.76 ± 0.02	0.96 ± 0.02	27 ± 5
		5:1	0.74 ± 0.03	0.95 ± 0.03	29 ± 6
	6	1:2	1.00 ± 0.04	1.06 ± 0.03	6 ± 5
		1.5:2	1.15 ± 0.04	1.19 ± 0.05	4 ± 5
		3:2	1.15 ± 0.07	1.20 ± 0.03	4 ± 7
		5:2	1.13 ± 0.05	1.22 ± 0.04	8 ± 6
Ex	2	-	0.90 ± 0.02	1.09 ± 0.03	22 ± 4
	3.7	-	0.89 ± 0.07	1.00 ± 0.05	13 ± 11

Model ink	**Min B/A mean value**	**Max B/A mean value**	**% increase**
1:1:1	0.98 ± 0.03	1.05 ± 0.03	7 ± 4
1.5:1:1	0.97 ± 0.06	1.05 ± 0.03	8 ± 7
1:1.5:1	0.92 ± 0.02	1.03 ± 0.05	13 ± 5
